# Immunopotentiation of Trivalent Influenza Vaccine When Given with VAX102, a Recombinant Influenza M2e Vaccine Fused to the TLR5 Ligand Flagellin

**DOI:** 10.1371/journal.pone.0014442

**Published:** 2010-12-28

**Authors:** H. Keipp Talbot, Michael T. Rock, Casey Johnson, Lynda Tussey, Uma Kavita, Anita Shanker, Alan R. Shaw, David N. Taylor

**Affiliations:** 1 Division of Infectious Diseases, Department of Medicine, Vanderbilt University Medical Center, Nashville, Tennessee, United States of America; 2 Division of Infectious Diseases, Department of Pediatrics, Vanderbilt University Medical Center, Nashville, Tennessee, United States of America; 3 Johnson County Clin-Trials, Lenexa, Kansas, United States of America; 4 VaxInnate Corporation, Cranbury, New Jersey, United States of America; 5 Health Decisions, Durham, North Carolina, United States of America; U.S. Naval Medical Research Center Detachment/Centers for Disease Control, United States of America

## Abstract

**Background:**

Currently controversy exists about the immunogenicity of seasonal trivalent influenza vaccine in certain populations, especially the elderly. STF2.4×M2e (VAX102) is a recombinant fusion protein that links four copies of the ectodomain of influenza virus matrix protein 2 (M2e) antigen to *Salmonella typhimurium* flagellin, a TLR5 ligand. The objectives of this study were to assess the feasibility of giving VAX102 and TIV in combination in an effort to achieve greater immunogenicity and to provide cross-protection.

**Methodology/Principal Findings:**

Eighty healthy subjects, 18-49 years old, were enrolled in May and June 2009 in a double-blind, randomized, controlled trial at two clinical sites. Subjects were randomized to receive either TIV + VAX102 or TIV + placebo. Both arms tolerated the vaccines. Pain at the injection site was more severe with TIV + VAX102. Two weeks after immunization the HAI responses to the H1 and H3 antigens of TIV were higher in those that received TIV + VAX102 than in TIV + placebo (309 vs 200 and 269 vs 185, respectively), although statistically non-significant. There was no difference in the HAI of the B antigen. In the TIV + VAX102 arm, the geometric mean M2e antibody concentration was 0.5 µg/ml and 73% seroconverted.

**Conclusions/Significance:**

The combination of TIV + VAX102 has the potential to increase the immune response to the influenza A components of TIV and to provide M2e immunity which may protect against influenza A strains not contained in seasonal TIV.

**Trial Registration:**

ClinicalTrials.gov NCT00921973

## Introduction

Influenza causes significant morbidity and mortality with an estimated 36,000 deaths annually in the US alone [1]. Vaccination is the primary method of prevention. Currently licensed vaccines require annual modifications since the vaccine is comprised of specific strain hemagglutinin (HA) glycoprotein of the influenza viruses anticipated to circulate in the coming year. In addition, concerns have arisen about the immunogenicity and protection provided by trivalent inactivated influenza vaccine (TIV) in populations such as the elderly [2]. Ideally, a vaccine that induces protective antibodies against viral structures of low or no variability could provide a constant level of long lasting immunity against influenza infection and provide sufficient immune stimulation to provide a protective response in the populations at highest risk for infection. One option is to use a vaccine with a genetically stable protein such as M2e along with TIV to increase immunogenicity and to provide greater cross-protection against other influenza A strains not represented in the seasonal vaccine.

The M2 protein of the influenza A virus ion channel is a non-glycosylated transmembrane protein that is expressed at high density in the cell membrane of viral infected cells and at low density in the lipid membrane of the mature influenza virus [3]. This protein undergoes little sequence variation, and antibodies to a component of the protein have provided significant protective activity in animal models [4]. M2e alone does not produce a significant immune response in humans, but does when presented as four tandem repeats genetically fused to flagellin, a TLR5 ligand [5]. This vaccine, designated VAX102 (STF2.4×M2e), was developed by VaxInnate Corporation as a cross-protective influenza A vaccine. The protein comprises *Salmonella typhimurium* flagellin type 2, or fljB, (STF2; TLR5 ligand) fused to four tandem repeats of M2e at the C-terminus of flagellin [5]. The M2e is similar to that of the M2e of the PR8 strain used in vaccine manufacturing except for 3 amino acid residues. It is produced as a fusion protein after purification in a prokaryotic fermentation system.

When STF2.4×M2e was injected with TIV into mice there was a 3 fold increase to the H1 component of TIV as measured by HAI compared to TIV alone (unpublished data). The stimulation of the innate immune system via the flagellin component of VAX102 appeared to be the possible mechanism to enhance the TIV response. Similarly, work using a synthetic TLR4 agonist given with TIV and an oil emulsion has shown greater IgG2a and IgG titers, higher HAI titers and type I cytokine responses in mice [6]. These results suggest that VAX102 when given with TIV in humans would enhance the immunogenicity of TIV as well as provide better cross-protection for circulating strains through immunity to M2e. This immunopotentiation to TIV would be desirable for populations like the elderly who respond less well to TIV [7]. The purpose of this study is to test the safety and immunogenicity of this novel adjuvant-antigen in combination with trivalent inactivated influenza vaccine in young healthy adults 18–49 years of age to determine if this combination is safe and able to immunopotentiate the response to TIV before testing in frail, elderly adults.

## Methods

### Study Design

This study, a phase I/II, double-blind, randomized, placebo-controlled trial, was designed to assess the safety, reactogenicity and immunogenicity of 1 µg of VAX102 investigational vaccine administered with the previous season's TIV, compared to placebo + TIV in healthy young adults at two clinical centers. The study was approved by the Institutional Review Boards at both sites (Vanderbilt University Medical Center and Johnson City Clin-Trials) and was conducted May-July 2009. The trial was registered with clinicaltrials.gov (NCT00921973). The protocol for this trial and supporting CONSORT checklist are available as supporting information; see Checklist S1 and Protocol S1.

### Participants

Forty subjects were randomized (1∶1 randomization) to each arm of the study. Subjects were considered eligible if they were age 18–49 years; able to adhere to all protocol requirements; healthy, as determined by medical history, physical examination, vital signs, and clinical safety laboratory examinations; and had not received influenza vaccination (TIV) during the 2008–2009 influenza season. Female subjects needed to have a negative urine pregnancy test within 24 hours preceding receipt of first vaccinations and needed to fulfill one of the following criteria: at least one year post-menopausal; surgically sterile; or willing to use a reliable form of contraception for the duration of the study.

Subjects were considered ineligible if they had any of the following: presence of significant acute or chronic, uncontrolled medical or psychiatric illness, history of cancer, impaired immunoresponsiveness (including diabetes or due to immunosuppressive treatment regimens), known hypersensitivity to a previous dose of influenza vaccine, allergy to eggs or any components of the study vaccines, receipt of influenza vaccination (TIV or LAIV) during the 2008–2009 influenza season, had known history of Guillain-Barré Syndrome, were vaccinated with a registered vaccine within 14 days (for inactivated vaccines) or 28 days (for live vaccines) prior to receiving the study vaccine, had a history of anaphylactic type reaction to injected vaccines, use of new prescription medications started within 7 days before study entry, receipt of any blood products, including immunoglobulin, within the 6 months before enrollment, or had clinical signs of active infection and/or oral temperature of ≥38°C (100.4°F).

### Ethics

The study was approved by the Vanderbilt Institutional Review Board and Mid*Lands IRB, Leawood, KS. Each subject signed a written consent.

### Interventions

Subjects were screened prior to enrollment to establish eligibility. Study materials were prepared by unblinded pharmacists and provided to blinded clinical staff. All study participants received Fluvirin 0.5 ml in the deltoid muscle of the non-dominant arm. Immediately following the Fluvirin injection, study participants received VAX102 1-µg dose (0.5 ml) vaccine or the identically appearing placebo by intramuscular injection at approximately the same vaccination site as the Fluvirin. Subjects remained in the clinic for 30 minutes after injection and returned to the clinic for safety observations on Days 1, 14 and 28 for a limited physical examination, memory aid review and laboratory assessment. Laboratory analysis included CBC, BUN, Creatinine, urinalysis and liver function tests.

Serum was drawn for hemagglutinin inhibition (HAI) antibodies and antibodies to M2e and flagellin on the day of vaccination and days 14 and 28 post-vaccination. In addition, blood was drawn to measure C-reactive protein (CRP) on the day of vaccination and the day following. Subjects maintained a memory aid to assess for reactogenicity and were evaluated one day post-vaccination and day 28 post-vaccination. Participants maintained the memory aid following vaccination, and for 6 days thereafter, on which they recorded solicited local and systemic reactions graded as none, mild, moderate, or severe. Adverse reactions were assessed by study participants using a 4 point scale (0–3) based on the interference of daily activities where 0 = no interference, 1 =  minimal interference, 2 =  moderate decreases in functioning, and 3 =  severe disruption. Solicited local reactions were redness, swelling or induration, pain and ecchymosis. Solicited systemic reactions were fever (subjects were provided with a thermometer), headache, joint pain, fatigue, muscle aches, shivering (chills) and increased sweating.

### Study Agents

#### Study Vaccine

VAX102 (STF2.4×M2e[Hu]) is comprised of 4 tandem copies of a consensus M2e sequence (4×M2e) fused to the TLR5-specific ligand *Salmonella typhimurium* flagellin fljB (STF) [5].

#### Licensed TIV

Fluvirin® (Novartis), an FDA licensed TIV for the 2008–2009 influenza season, was used in all participants. Each 0.5-mL dose contained a total of 15 µg of influenza virus hemagglutinin (HA) from each of the following 3 strains: A/Brisbane/59/2007(H1N1); A/Uruguay/716/2007 (H3N2), an A/Brisbane/10/2007-like strain; and B/Florida/4/2006.

#### Placebo

The placebo used in this study was the buffer used to formulate VAX102. This buffer, designated F105, contains 10 mM Tris, 10 mM Histidine, 5% (w/v) sucrose, 75 mM NaCl, 0.1 mM EDTA, 0.5% (v/v) ethanol and 0.02% (w/v) polysorbate-80 at pH 7.2.

### Laboratory Assays

#### HAI

Hemagglutination inhibition (HAI) antibody titers for both influenza A and B vaccine antigens were determined in duplicate (on separate days), with paired specimens tested simultaneously. HAI antibodies were determined using CDC protocols [8] using 0.5% turkey red blood cells with representative antigens supplied by the CDC. Serum samples were treated per manufacturer's instruction with receptor-destroying enzyme (RDE) before testing. HAI assays were performed at a starting dilution of 1∶10 with subsequent serial 2-fold dilutions. Each antigen (A/Brisbane/59/2007, A/Brisbane/10/2007, and B/Florida/4/2006) was diluted to 8 HA units. Samples showing high background in testing were repeated after preabsorption with packed turkey red blood cells. Titers were determined by identifying the last well with total lack of agglutination. Samples where duplicate values were greater than 2-fold different were repeated. Samples with values greater than the last dilution were diluted further and retested. Final results were the reciprocal titer of the lowest duplicate value.

#### M2e

M2e-specific IgG levels were determined by ELISA. The M2e peptide is identical to the sequence used in the VAX102 vaccine (STF2.4×M2e—SLLTEVETPIRNEWGSRSNDSSDP). The peptide was coated on the plates at 2 µg/mL. After overnight incubation at 2–8°C, plates were washed and blocked (Superblock with Tween 20, Pierce, Rockford, IL, USA). Dilutions of subject serum were prepared in a separate plate and transferred to M2e-coated plates. After incubation and washing, plates were developed using goat anti-human IgG conjugated with horseradish peroxidase (HRP) (Jackson Immunochemicals, West Grove, PA, USA), TMB substrate (Pierce One Step, Rockford, IL, USA) and H_2_SO_4_ stop solution. Plates were read at 450 nm. Adjusted results were calculated for each subject and bleed date. A sample with an adjusted result of ≥0.174 µg/mL was considered positive. The binding of human serum samples to M2e-coated plates was compared to a standard curve of human polyclonal IgG. The curve was fitted using a 4-parameter logistic equation in Softmax Pro 5.2 (Molecular Devices, Sunnyvale, CA, USA). Pooled positive and negative control sera were run on each plate. Results of subject and control serum were converted from OD values to M2e-specific IgG using the standard curve and adjusting for dilution. Pass/fail criteria for each assay were established based on both the standard curve performance and the adjusted results of the positive and negative serum. The human IgG (AbD Serotec, Raleigh, NC, USA) was bound to plates (Immulon 4 HBX, Thermo Fisher Scientific Inc, Waltham, MA, USA) at 4-fold dilutions starting at 3.6 µg/mL.

#### Flagellin (STF2)

The STF2 protein was identical to the sequence used in the VAX102 vaccine (STF2.4×M2e). This protein was coated on the plate at 1 µg/mL. After overnight incubation at 2–8°C, plates were washed and blocked (Superblock with Tween 20, Pierce, Rockford, IL, USA). Dilutions of subject serum were prepared in a separate plate and transferred to STF2-coated plates. After incubation and washing, plates were developed with goat anti-human IgG conjugated HRP (Jackson Immunochemicals, West Grove, PA, USA), TMB substrate (1-Step, Pierce, Rockford, IL, USA) and H_2_SO_4_ stop solution. Plates were read at 450 nm and adjusted results were calculated for each subject and time point. For the STF2 ELISA, the binding of human serum samples to STF2-coated plates was compared to a standard curve of human polyclonal IgG. The curve was fitted as described above. Pooled positive control sera were run on each plate. Results of subject and control serum were converted from OD values to STF2-specific IgG using the standard curve and adjusted for dilution. The human IgG (AbD Serotec, Raleigh, NC, USA) was bound to plates (Immulon 4 HBX, Thermo Fisher Scientific, Waltham, MA, USA) at 4-fold dilutions starting at 3.6 µg/mL.

### Objectives

The primary objective of the study was to assess the safety, reactogenicity, and tolerability of VAX102 when given with Trivalent Inactivated Influenza Vaccine (TIV) delivered in the same arm as two separate IM injections in healthy adults 18 to 49 years. Secondary objectives included the assessment of the immunogenicity of the VAX102 when given with TIV and the antibody response to TIV when given with VAX102 compared to TIV alone.

### Outcomes

Safety assessment included visual assessment of the injection site on day of administration (predose) and at 30 minutes post-dose and on Days 1, 14 and 28; solicitation of local and systemic reactogenicity; and local and systemic reactogenicity reporting in the 7 days after vaccination. The immunogenicity assessment was determined by serum IgG responses to M2e and HAI was assessed before vaccination on Day 0 and on Days 14 and 28 after vaccination.

### Sample Size and Randomization

Selection of sample size was based on a calculation using 80% power, a type I probability of 0.05, and a 40% higher rate of seroconversion of the TIV + VAX102 arm compared to the TIV + placebo arm. The study was not powered to detect a difference of less than 40%. The randomization was done by block randomization, block size fixed at 4, and was stratified by clinical site. Eligible subjects were assigned to an arm according to a randomization code provided by an independent statistician. The independent statistician provided the randomization information to the investigational pharmacist or designee. Investigational vaccine was prepared by an unblinded investigational pharmacist who was not involved in vaccine administration or subsequent clinical assessments. Sponsor personnel involved in generation and recording of immunogenicity assays were blinded until all assays and repeats were completed and final data sets were provided to data management personnel and locked in the database.

### Statistical Analyses

The study hypothesis was that VAX102 vaccine when administered with TIV, intramuscularly, would be generally well-tolerated and would elicit M2e and HAI antibodies greater than, those elicited by the placebo with TIV delivered intramuscularly. Immunogenicity parameters included the geometric mean of pre- and post-vaccination anti-M2e and HAI serum antibody concentrations and the proportion of subjects with an M2e and HAI specific antibody after vaccination. In general, categorical variables were summarized by study arm as frequencies and percentages in each category. Continuous variables were summarized by study arm as numbers of subjects, means, standard deviations, medians, and minimum/maximum values. Wilcoxon rank sum test was used to calculate p-values for the geometric means. Statistical analyses were performed at the two-sided significance level of α = 0.05 unless otherwise stated. No adjustments were made for multiple statistical testing. All programs for data output and analyses were written in SAS® version 9.1 (SAS Institute, Cary, NC) and STATA^TM^ version 9 (StataCorp, College Station, TX).

All analyses were based upon a per-protocol cohort with additional analysis performed for the intent-to-treat (ITT) cohort. The per-protocol cohort is defined as all volunteers who completed immunization. The primary immunogenicity population consisted of all subjects who received immunization and had baseline and post-baseline anti-M2e serum antibody or HAI titers. Safety and tolerability analyses included all subjects and included descriptive statistics. Frequency of vaccination site abnormalities; incidence of local and systemic AEs and their relationship to the study drug; and changes in clinical laboratory results, vital signs, and physical examination findings were the primary safety measures.

Anti-M2e or HAI serum antibody titers were listed and summarized for each time point that data were available. The mean change from baseline to Day 14 and 28 (±2) in the log-transformed anti-M2e or HAI serum antibody reciprocal titers were compared to zero within treatment arms using t-tests and the data was assumed to be log-normally distributed. The 95% CI for the geometric mean was calculated by deriving the 95% CI around the mean on the log scale and then taking the antilog of the confidence interval on the log scale. HAI results were also stratified by gender. Seroprotection was defined as a post-vaccination HAI titer of ≥1∶40 and seroconversion was defined either as an initial titer <10 and a post-vaccination titer ≥40 or as an initial titer ≥10 and a four-fold rise or greater increase in titer compared to baseline. Seroconversion for M2e was defined as having a 4-fold or greater increase in anti-M2e antibody concentration compared to baseline and having a post-baseline value ≥0.174 µg/mL of IgG.

## Results

A total of 80 subjects were enrolled (40 at each site) during May and June 2009. All subjects completed the protocol through day 14. 39 subjects in the VAX102 + TIV arm and 36 in the placebo + TIV arm completed through day 28. Subjects were similar except for a higher proportion of males in the TIV + placebo arm (Table 1).

**Table 1:** Baseline characteristics of the study subjects according to study arm comparing standard trivalent influenza vaccine and placebo to TIV and VAX102 (STF2.4×M2e).

Characteristic	TIV + placeboN = 40 (%)	TIV + VAX102N = 40 (%)
**Gender** (% male)	20 (50)	12 (30)
**Race**WhiteBlackOtherEthnicity (% Hispanic)	30 (75)7 (17.5)3 (7.5)0 (0)	28 (70)11 (27.5)1 (2.5)3 (7.5)
**Age**Mean (years)Median (range, years)	29.826.5 (18–49)	28.225.0 (18–48)
**Received influenza vaccine in last 5 years**	12 (30)	13 (32.5)

### Immunogenicity

**Figure 1:**
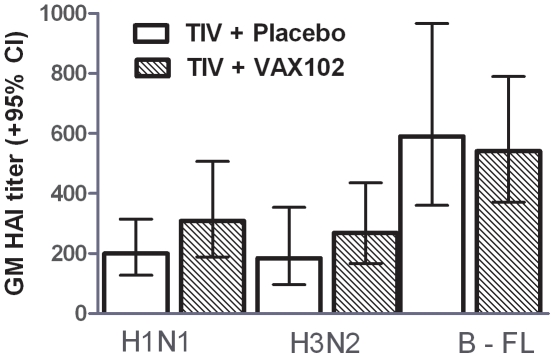
Comparison of Geometric Mean HAI Titers among subjects who received TIV +placebo or TIV plus VAX102.

The HAI pre-vaccination geometric mean titers (GMT) for all three TIV antigens were similar between both arms (table 2). The participants in the VAX102 + TIV arm had approximately a 1.5 fold greater response to those in the placebo + TIV arm for both H1N1 and H3N2 on day 14 but this was not statistically significant (table 2, figure 1). These results were consistent at the two clinical sites. Table 3 shows the HAI responses stratified by gender for each study arm. The greatest fold change associated with the VAX102 vaccine was seen in the men. Table 4 also shows the seroprotection and seroresponse at Days 14 and 28 post-vaccination with similar results seen in both arms.

**Table 2:** HAI Geometric Mean Immunologic Responses among subjects who received one dose of TIV co-administered with placebo or VAX102 as a separate injection at the same anatomical location.

HAI		TIV + placebo		TIV + VAX102		
		N = 40		N = 40		
Vaccine Strain	Days Post-vaccination	GMT^†^	95% CI	GMT^†^	95% CI	Ratio
H1N1	01428	12200165	8, 19127, 31497, 281	16309240	10, 23188, 506147, 392	1.541.46
H3N2	01428	11184151	8, 1696, 35376, 297	9269184	7, 12166, 435114, 297	1.461.22
B	01428	46590458	26, 82360, 966252, 832	60541514	39,94371, 789349, 756	0.921.12

GMT  =  Geometric Mean Titer.

**Table 3:** CRP and HAI Geometric Mean Immunologic Responses among subjects who received one dose of TIV co-administered with placebo or VAX102 as a separate injection at the same anatomical location, stratified by gender for each study arm.

	TIV + Plac	TIV + M2e		TIV + Plac	TIV + M2e	
SEX	Males	Males		Females	Females	
Subject	n = 20	n = 12	Ratio	n = 20	n = 28	Ratio
CRP D0	0.9	0.6	0.7	1.1	2.0	1.8
CRP D1	0.9	6.4	7.3	1.3	10.4	7.9
CRP Fold	1.0	10.4	10.4	1.2	5.1	4.4
H1N1 d0	12	15	1.2	13	16	1.3
H1N1 d14	144	359	2.5	279	290	1.0
H1N1 d28	104	300	2.9	240	221	0.9
H1 fold d14	12	24	2.0	22	18	0.8
H1 fold d28	10	21	2.2	19	13	0.7
H3N2 d0	8	11	1.4	17	9	0.5
H3N2 d14	113	214	1.9	302	297	1.0
H3N2 d28	80	150	1.9	251	200	0.8
H3 fold d14	14	20	1.4	18	33	1.8
H3 fold d28	10	17	1.7	15	22	1.5
B-FL d0	49	53	1.1	44	64	1.4
B-FL d14	590	570	1.0	591	530	0.9
B-FL d28	553	497	0.9	394	521	1.3
B fold d14	12	11	0.9	13	8	0.6
B fold d28	13	9	0.7	9	8	0.9

**Table 4:** Seroprotection (SP) and seroresponse (SR) rates among subjects who received one dose of TIV co-administered with placebo or VAX102 as a separate injection at the same anatomical location.

VaccineStrain	Dayspost-vaccination	TIV + placebo		TIV + VAX102	
		% SP	% SC	% SP	% SC
	0	22.5		35	
H1N1	14	92.5	75	92.5	82.5
	28	88.9	77.8	92.3	76.9
	0	20		12.5	
H3N2	14	82.5	80	90	90
	28	77.8	75	87.2	87.2
	0	65		75	
B	14	97.5	82.5	95	75
	28	94.4	69.4	97.4	74.4

Seroprotection is post-vaccination HAI titer of ≥1∶40.

Seroresponse is either an initial HAI <10 and a post vaccination titer ≥40 or an initial titer ≥10 and a 4-fold or greater increase in titer compared to baseline.

In the arm that received the combination of VAX102 + TIV 29 (73%) of 40 subjects seroconverted to M2e after a single dose (Table 5). The M2e antibody concentration was very low at baseline (0.04) and rose to 0.52 post-vaccination, an approximately 10-fold increase. The flagellin antibody titers increased from 0.74 to 27, a more than a 30-fold increase. The CRP was measured pre-vaccination on day 0 and on the following day. There was a minimal increase in CRP in the arm that received TIV + placebo while there was a mean 6-fold increase among subjects who received TIV + VAX102 (Table 5).

### Reactogenicity

In general the vaccinations were well tolerated among the 80 subjects. According to patient self-report, none of the subjects in either arm had fever. In addition, temperature was measured in the clinic on days 1, 14 and 28 and those were within normal range. Most local and systemic symptoms were mild or moderate in severity (Table 6). Nearly all subjects had some degree of pain at the site of injection. Most were mild in TIV + placebo arm and most were moderate in the TIV + VAX102 arm. Three subjects (7.5%) in the VAX 102 + TIV arm had severe injection pain. Redness and swelling at the site of injection were not reported in the TIV + placebo arm compared to about 20% of the subjects reported mild to moderate redness and swelling with TIV + VAX102. Systemic symptoms occurred infrequently in both arms. Muscle aches were the most common occurring in 7.5% in the placebo arm and 37.5% in the VAX102 arm. In the placebo arm, 5 subjects had an adverse event, all mild or moderate, and all were considered unrelated to vaccine. In the VAX102 arm, 8 subjects had an adverse event, all mild to moderate. Only one of these, injection site erythema, was considered to be due to the injection.

**Table 5:** Geometric Mean Concentration (GMC) Antibody Response to M2e and flagellin and CRP response after one dose of VAX102.

	TIV + Placebo	TIV + M2e	
Days post-vaccination	N = 40*	N = 40**	p-value
**M2e antibody response**			
Day 0	0.03	0.04	
Day 14	0.04	0.52	0.007
Day 28	0.04	0.46	0.013
Geo. Mean fold rise day 14	1.06	11.9	
Geo. Mean fold rise day 28	1.1	9.99	
Seroconversion rate†	0	29 (73%)	
**Flagellin antibody response**			
Day 0	0.52	0.74	
Day 14	0.51	26.78	0.0002
Day 28	0.55	21.97	0.0023
Geo. Mean fold rise day 14	0.98	36.25	
Geo. Mean fold rise day 28	1.04	29.74	
**CRP value**			
Day 0	1	1.41	
Day 1	1.08	8.97	<0.0001
Geo. Mean fold rise	1.08	6.35	

*For Day 28 values N = 36.

**For Day 28 values N = 39.

† Seroconversion to M2e was defined as a serum IgG anti-M2e antibody value ≥0.174 µg/ml and a four-fold rise in titer.

**Table 6:** Solicited local and systemic reactogenicity within 7 days of either trivalent influenza vaccine (TIV) and placebo or TIV and VAX102 given in the same arm.

Symptom	Severity	TIV + placeboN = 40 (%)	TIV + VAX102N = 40 (%)
Fever	MildModerateSevere	0 (0)0 (0)0 (0)	0 (0)0 (0)0 (0)
Redness	MildModerateSevere	0 (0)0 (0)0 (0)	1 (2.5)8 (20)0 (0)
Swelling	MildModerateSevere	0 (0)0 (0)0 (0)	4 (10)3 (7.5)0 (0)
Bruising	MildModerateSevere	0 (0)0 (0)0 (0)	1 (2.5)0 (0)0 (0)
Injection Site Pain	MildModerateSevere	11 (27.5)3 (7.5)0 (0)	8 (20)24 (60)3 (7.5)
Headache	MildModerateSevere	5 (12.5)2 (5)0 (0)	10 (25)3 (7.5)1 (2.5)
Fatigue	MildModerateSevere	3 (7.5)2 (5.0)0 (0)	7 (17.5)2 (5.0)0 (0)
Joint Pain	MildModerateSevere	0 (0)1 (2.5)0 (0)	1 (2.5)0 (0)0 (0)
Muscle Ache	MildModerateSevere	2 (5.0)1 (2.5)0 (0)	9 (22.5)5 (12.5)1 (2.5)
Shivering/Chills	MildModerateSevere	0 (0)0 (0)0 (0)	1 (2.5)0 (0)0 (0)
Increased Sweating	MildModerateSevere	1 (2.5)1 (2.5)0 (0)	0 (0)1 (2.5)0 (0)

## Discussion

This study was a phase I/II to evaluate the safety and immunogenicity of an M2e protein influenza vaccine when given with seasonal TIV in young healthy adults. Although limited by sample size, this study did show a statistically non-significant increase of approximately 1.5 fold in the GMT of the HAI response to both H1N1 and H3N2. In contrast there was little difference in the influenza B component. The immune response to influenza B was the highest compared to the other antigens and it is unlikely that they could be further enhanced with the addition VAX02. Further, in contrast to the 24 residue M2 ectodomain of influenza A, the M2 ectodomain of influenza B has only 7 residues. [9] Thus it is unlikely that A/M2e could enhance the immune response of influenza B. The seroprotection and seroconversion rates were similar for both arms but this is likely due to the high baseline titers in both arms (likely due to a history of prior vaccination and infection) and due to the historically good responses seen to vaccination in this age group compared to adults ≥65 years of age. [7] In a recent study by Falsey et al. [10] a high dose TIV with a 4-fold increase in HA content was only able to induce a 1.7 fold increase in H1N1 or H3N2 titer in older adults. The current study, performed in young adults, produced a similar rise of 1.5 fold. Interestingly the greatest fold change was seen in men. Two factors appeared to account for this difference. One was that females had a 2–3 fold higher response than males. It appears that if the response is poor than M2e can enhance the response, but if the response is strong the addition of M2e does not add much. Gender differences have been previously seen with pertussis vaccination where women had a slightly lower antibody response than men [11].

VAX102 also produced an immune response to M2e which was not noted in the placebo arm. Even though the M2e component of VAX102 has a difference of 3 amino acids compared to the PR8 virus which is used for vaccine manufacturing, a prior study has shown that mice are able to generate immune serum that will react to M2e from multiple different origins. [12] Antibodies to M2e are protective in animal models, [4], [5] but there are no clinical studies testing the efficacy of M2e or any estimates of immunologic correlates of protection. Future studies will need to determine the level of additional protection provided by anti-M2e antibody and increased HAI antibody titers. CRP responses were elevated in the arm that was immunized with TIV + VAX102 but not in the arm that received TIV + placebo. CRP rises when interleukin-6 is elevated which is most likely due to the stimulation of the TLR5 receptor by flagellin. [13] TIV alone does not induce this type of response. The stimulation of the innate immune system by flagellin is what induces the M2e antibody response and appears also to enhance the response to the hemagglutinin component of TIV. The M2e component of VAX102 is derived from the consensus sequence of human influenza A strains. It is interesting that VAX102 increased the immune response to the influenza A components of TIV, namely the H1 and H3 components but did not improve the B component. The response to the B strain was the highest of all three antigens and it is possible that it was maximally stimulated.

Due to the concerns about the effectiveness of the current TIV in older adults, Poland et al. [2] summarized the available and previously tested influenza vaccines and influenza vaccine strategies to improve the effectiveness of the current TIV. The combination of VAX102 + TIV would be another possibility for an improved vaccine formulation. The increased immunogenicity and addition of greater strain cross-protection would theoretically increase the number of senior citizens protected from influenza.

This preliminary work has shown the VAX102 when used in combination with TIV to have the potential to improve the immunologic response to TIV alone. This combination may have promise in high-risk populations such as the elderly.

## Supporting Information

Checklist S1 CONSORT Checklist.
(0.22 MB DOC)

Protocol S1 Trial Protocol.
(0.36 MB PDF)
