# Supplementary material for: Immunopotentiation of Trivalent Influenza Vaccine When Given with VAX102, a Recombinant Influenza M2e Vaccine Fused to the TLR5 Ligand Flagellin
Source: PLoS One. 2010 Dec 28;5(12):e14442. doi: 10.1371/journal.pone.0014442 (PMC3010987; doi:10.1371/journal.pone.0014442)
Supplement: Protocol S1 — Trial Protocol. (0.36 MB PDF) [file pone.0014442.s002.pdf]

## PROTOCOL VAX102-07

# A Multicenter, Double-Blinded, Randomized, Placebo Controlled Study to Investigate the Safety and Immunogenicity of VAX102 when given in the same arm with the Standard Influenza Vaccine in Healthy Adults

**Investigational Product:** VAX102 [STF2.4xM2e(Hu)]

**Sponsor:**

VaxInnate Corporation
3 Cedar Brook Drive
Cranbury, NJ 08540
609-860-2260

**Sponsor's Study Director:**

David N. Taylor, MD
Chief Medical Officer
VaxInnate Corporation
3 Cedar Brook Drive
Cranbury, NJ 08512
Voice 609-860-2260, Fax 609-860-2290

**Version / Date:** 2.0 / 28 April 2009

The information in this study protocol is strictly confidential and is available for review to Investigators, study center personnel, the ethics committee, and the health authorities. It will not be disclosed to third parties without written authorization from the Sponsor, except to obtain informed consent from persons receiving the study treatment. Once the protocol is signed, its terms are binding for all parties.

**CONFIDENTIAL**

# STATEMENT OF COMPLIANCE

The study will be carried out in accordance with Good Clinical Practice (GCP) as required by the following:

- United States (US) Code of Federal Regulations (CFR) applicable to clinical studies (45 CFR Part 46; 21 CFR Part 50, 21 CFR Part 56, and 21 CFR Part 312)
- ICH E6 and 62 Federal Register 25691 (1997): Good Clinical Practice (GCP): Consolidated Guideline

# SPONSOR'S SIGNATURE PAGE

The signature below constitutes the approval of this protocol and the attachments, and provides the necessary assurances that this trial will be conducted according to all stipulations of the protocol, including all statements regarding confidentiality, and according to local legal and regulatory requirements and applicable US federal regulations and ICH guidelines.

Principal Investigator: ______________________________________________________

Date

## TABLE OF CONTENTS

**TABLE OF CONTENTS ..... 3**

**1 INTRODUCTION ..... 12**

**2 OBJECTIVES ..... 16**

**3 STUDY DESIGN ..... 17**

**4 STUDY POPULATION ..... 17**

4.1 Number of Subjects ..... 17
4.2 Inclusion Criteria ..... 17
4.3 Exclusion Criteria ..... 18
4.4 Subject Enrollment and Randomization ..... 19
4.5 Unblinding ..... 20
4.6 Withdrawal of Subjects from the Study ..... 20
4.7 Halting Rules ..... 21

**5 STUDY PRODUCT DESCRIPTION ..... 21**

5.1 Investigational Product ..... 21
5.1.1 VAX102 (STF2.4xM2e[Hu]) ..... 21
5.1.2 Trivalent Inactivated Influenza Vaccine (TIV) ..... 21
5.1.3 Placebo (F105 – Formulation Buffer) ..... 22
5.2 Storage and Handling ..... 22
5.3 Investigational Product Preparation ..... 22
5.3.1 VAX102 Vaccine Dilution Scheme Group A Dose 1.0 µg for 0.5 mL volume for IM Injection ..... 23
5.3.2 Placebo (F105) Group B for 0.5 mL volume for IM Injection ..... 23
5.3.3 TIV Vaccine for Groups A-B STD for 0.5 mL volume for IM Injection ..... 23
5.4 Investigational Product Administration ..... 23
5.5 Accountability of Investigational Products ..... 24

**6 STUDY SCHEDULE ..... 24**

6.1 Screening Visit (day -30 to -1) ..... 24
6.2 Dosing and Early Follow-up ..... 24
6.2.1 Day 0 Vaccination ..... 24
6.2.2 Day 1 (+1) ..... 25
6.2.3 Day 14 (±2) ..... 26
6.2.4 Day 28 (±2) ..... 26
6.2.5 Unscheduled Visit ..... 26

**7 STUDY PROCEDURES AND EVALUATIONS ..... 27**

7.1 Clinical Procedures and Evaluations ..... 27
7.1.1 Screening ..... 27
7.1.2 Medical History and Physical Examination ..... 27

7.1.3 Targeted Physical Examination, Baseline Signs and Symptoms, and Injection Site Assessment, and Vital Signs Before and/or After Vaccination ............ 28
7.1.4 Safety and Tolerability Evaluation ............ 28
7.1.5 Concomitant Medications ............ 28
7.2 Laboratory Evaluations ............ 28
7.2.1 Clinical Laboratories ............ 28
7.2.2 Specific Antibody Assays ............ 29
7.2.3 Specimen Collection, Preparation, Handling, and Shipping ............ 29

**8 ASSESSMENT OF SAFETY ............ 30**

8.1 Definition of Immediate Complaints, Vaccine Reactogenicity and Adverse Events 30
8.1.1 Immediate Complaints ............ 30
8.1.2 Vaccine Reactogenicity ............ 30
8.1.3 Adverse Events ............ 30
8.1.4 Clinical Laboratory Findings as Adverse Events ............ 31
8.1.5 Double Reporting of Reactogenicity Findings/Complaints ............ 32
8.2 Definition of Serious Adverse Events ............ 33
8.3 SAE Reporting Procedures ............ 33
8.3.1 SAE Detection and Reporting ............ 33
8.3.2 Reporting of Pregnancy ............ 34
8.3.3 Procedures to be Followed in the Event of Abnormal Laboratory Test Values or Abnormal Clinical Findings ............ 35
8.3.4 Type and Duration of Follow-up of Subjects after Adverse Events ............ 35
8.4 Halting Rules ............ 35
8.5 Safety Oversight ............ 35

**9 DATA COLLECTION, PROCESSING AND ANALYSIS ............ 36**

9.1 Data collection and processing ............ 36
9.2 Statistical methods ............ 36
9.2.1 Overview and Study Objectives ............ 36
9.2.2 Sample size and power ............ 37
9.2.3 Analysis populations ............ 37
9.2.4 Study Hypothesis ............ 37
9.2.5 General statistical analysis methods ............ 37
9.2.6 Baseline characteristics ............ 37
9.2.7 Study drug administration ............ 37
9.2.8 Concomitant medications ............ 37
9.2.9 Immunogenicity analysis ............ 38
9.2.10 Safety analysis ............ 38
9.2.10.1 Subject disposition ............ 38
9.2.10.2 Reactogenicity and other AEs ............ 39
9.2.10.3 Clinical laboratory evaluation ............ 39
9.2.10.4 Vital signs ............ 40
9.2.10.5 Physical examination and Injection Site Evaluation ............ 40

**10 CLINICAL STUDY MONITORING AND ADMINISTRATION ............ 40**

10.1 Informed Consent........................................................................... 40
10.2 Study Documentation.................................................................... 40
10.3 Investigator Brochure.................................................................... 41
10.4 Confidentiality and Publication ...................................................... 41
10.5 Institutional Review Board ............................................................. 42
10.6 Ethical Study Conduct .................................................................... 42
10.7 Study Monitoring ............................................................................ 42
10.8 Quality Control and Quality Assurance ......................................... 43
10.9 Protocol Amendments .................................................................... 43

**11 REFERENCES.................................................................................................. 44**

**APPENDIX A. SOLICITED LOCAL AND GENERAL AES ............................................ 45**

**APPENDIX B. TOXICITY GRADING SCALE FOR HEALTHY ADULT AND ADOLESCENT VOLUNTEERS ENROLLED IN PREVENTIVE VACCINE CLINICAL TRIALS........................................................................................................ 46**

A Tables for Clinical Abnormalities.................................................... 46
B Tables for Laboratory Abnormalities............................................... 47

**APPENDIX C: SCHEDULE OF PROCEDURES AND ASSESSMENTS FOR VACCINATION ........................................................................................................ 49**

**LIST OF TABLES**

Table 1. Study design for VAX102-02 ........................................................... 13
Table 2. Local and systemic symptoms after the first dose of VAX 102-01/102-02 or placebo ............................................................................................ 13
Table 3. Number of volunteers listed by route of administration and dose of VAX102............. 14
Table 4. CRP results listed by route of administration and dose………………………………..15
Table 5. Enhancement of H1 HAI with concomitant administration of VAX102 with Flulaval in mice…………………………………………………………..…………………
…… 16
Table 6. Number of volunteers listed by dose and investigational vaccine …………………… 17

# LIST OF ABBREVIATIONS

| | | | |
|---|---|---|---|
| AE | Adverse event | IgG | Immunoglobulin G |
| ALT | Alanine aminotransaminase | IgM | Immunoglobulin M |
| ANOVA | Analysis of variance | IM or i.m. | Intramuscular |
| AST | Aspartate aminotransaminase | IND | Investigative New Drug application |
| b-HCG | Beta – human chorionic gonadotropin | IRB | Institutional review board |
| BP | Blood pressure | ITT | Intent to treat |
| BUN | Blood Urea Nitrogen | IUD | Intrauterine device |
| °C | Degrees Celsius | IV | Intravenous |
| CBC | Complete Blood Count | LLT | Lowest Level Term |
| CBER | Center for Biologics Evaluation and Research, FDA | M2e | Influenza A M2 ectodomain antigen |
| CFR | Code of Federal Regulations | MedDRA® | Medical Dictionary for Regulatory Activities |
| Cm | Centimeters | mEq/L | Milliequivalents per liter |
| CRF | Case Report Form | mg | Milligram |
| CRP | C-reactive Protein | mm | Millimeter |
| CMH | Cochran-Mantel-Haenszel | mm Hg | Millimeter mercury (re: blood pressure) |
| DDC’c | Dendritic cells | N | Number (typically refers to subjects) |
| Dl | Deciliter | NA | Neuraminidase |
| ELISA | Enzyme-linked immunosorbent assay | PI | Principal Investigator |
| ER | Emergency Room | PT | Preferred Term |
| °F | Degrees Fahrenheit | µg | Microgram |
| FDA | Food and Drug Administration | RR | Respiratory Rate |
| FLA | Flagellin | SAE | Serious adverse event |
| GCP | Good clinical practice | SC or s.c. | Subcutaneous |
| Gm | Gram | SGPT | Serum glutamic pyruvic transaminase |
| GLP | Good laboratory practice | SGOT | Serum glutamic oxaloacetic transaminase |
| GMP | Good manufacturing practice | SI | Solomon Islands |
| HA | Hemagglutinin | SID | Subject Identification Number |
| HAI | Hemagglutination inhibition assay | SMC | Safety Monitoring Committee |
| HEENT | Head, ears, eyes, nose and throat examination | SOC | System Organ Class |
| HBsAg | Hepatitis B surface antigen | SOP | Standard Operating Procedure |
| Hct | Hematocrit | STF2 | Salmonella typhimurium flagellin type 2 |
| Hgb | Hemoglobin | TIV | Trivalent Influenza vaccine |
| HR | Heart Rate | TLR | Toll-like receptor(s) |
| HRP | Horse radish peroxidase | ULN | Upper limit of normal |
| ICF | Informed Consent Form | VAX102 | STF2.4xM2e(Hu) Influenza A M2e vaccine |
| ICH | International Conference on Harmonization | VAX125 | STF2.HA1(SI) Influenza A H1 SI vaccine |
| ID | Intradermal | WBC | White Blood Cell Count |
| IgA | Immunoglobulin A | WHOdd | World Health Organization Drug Dictionary |

# PROTOCOL SYNOPSIS

| | |
|---|---|
| **STUDY NUMBER** | VAX102-07 |
| **STUDY TITLE** | A Multicenter, Double-Blinded, Randomized, Placebo Controlled Study to Investigate the Safety and Immunogenicity of VAX102 when given in the same arm with the Standard Influenza Vaccine in Healthy Adults |
| **SPONSOR** | VaxInnate Corporation |
| **PHASE** | II |
| **STUDY POPULATION** | Healthy adult volunteers age 18 to 49 years inclusive |
| **NUMBER OF SITES** | 2 sites |
| **STUDY DURATION** | The study duration for each subject will be approximately 4 weeks following administration of the dose of investigational vaccine (total study duration approximately 8 weeks). |
| **INVESTIGATIONAL PRODUCT** | STF2.4xM2e(Hu) (VAX102), which is a recombinant fusion protein that links four tandem copies of the influenza A virus M2e antigen to the C-terminus of *Salmonella. typhimurium* flagellin, a TLR5 ligand.<br>The Trivalent Inactivated Influenza Vaccine (TIV) will contain 15 mcg of hemagglutinin antigen for each of the contemporary influenza strains. The 2008-2009 Northern hemisphere vaccine strains are:<br>A/Brisbane/59/2007 (H1N1)-like, A/Brisbane/10/2007 (H3N2)-like, and B/Florida/4/2006-like antigens |
| **CONTROL PRODUCT** | Buffer (F105) that contains 10 mM Tris, 10 mM Histidine, 5% (w/v) sucrose, 75 mM NaCl, 0.1 mM EDTA, 0.5% (v/v) ethanol and 0.02% (w/v) polysorbate-80 at pH 7.2 |
| **REGIMEN AND DOSING** | Intramuscular (IM) vaccination with 1 µg of STF2.4xM2e(Hu) (VAX102) with TIV or F105 (Placebo) with TIV to be given Day 0. |
| **OBJECTIVES** | **Primary:** To assess the safety, reactogenicity, and tolerability of VAX102 when given with Trivalent Inactivated Influenza Vaccine (TIV) delivered in the same arm as two separate IM injections in healthy adults 18 to 49 years.<br>**Secondary:**<br>• To assess the immunogenicity of the VAX102 when given with TIV.<br>• To assess the antibody response to TIV when given with VAX102 compared to TIV alone. |
| **STUDY DESIGN** | A multicenter, double-blind, randomized, placebo controlled vaccine study to assess the safety, reactogenicity and immunogenicity of one subject group receiving 1 µg VAX102 investigational vaccine with TIV, delivered intramuscularly into the nondominant arm as a prime vaccination, compared to placebo with TIV, in healthy normal adults of |

both genders. All subjects will receive TIV first followed by VAX102 or Placebo and undergo identical study procedures.

Eligible subjects will be assigned to a group according to a randomization code provided. The randomization is 1:1 for each of the 2 different groups and summarized as follows:

**Number of volunteers listed by dose and investigational vaccine**

| Groups | Vaccine 1 | Vaccine 2 | Total # Subjects |
|---|---|---|---|
| **A** | TIV (std dose) | VAX102 1 μg | 40 |
| **B** | TIV (std dose) | Placebo | 40 |
| **Total** | | | 80 |

Dosing will occur on Day 0.

**SAMPLE SIZE**

Up to 80 healthy adult volunteers of both genders

**INCLUSION/ EXCLUSION CRITERIA**

INCLUSION

- Male or female aged 18 – 49 years inclusive
- Give written informed consent to participate in the study and adherence to all protocol requirements.
- Healthy, as determined by medical history, physical examination, vital signs, and clinical safety laboratory examinations
- Females should fulfill one of the following criteria:
    1. At least one year post-menopausal,
    2. Surgically sterile,
    3. Will use oral, implantable, transdermal or injectable contraceptives for 30 days prior to vaccination and for the duration of the study
    4. Willing to use reliable form of contraception approved by the Investigator (*e.g.*, intrauterine device (IUD), female condom, diaphragm with spermicide, cervical cap, use of condom by the sexual partner or a sterile sexual partner) for the duration of the study.
- Women of childbearing potential must have a negative urine pregnancy test within 24 hours preceding receipt of vaccination.
- Comprehension of the study requirements, expressed availability for the required study period, and ability to attend scheduled visits.
- Did not receive influenza vaccination (TIV) during the 2008-2009 influenza season.

EXCLUSION

- Presence of significant acute or chronic, uncontrolled medical or psychiatric illness (institution of new medical or surgical treatment, or a significant dose alteration for uncontrolled symptoms or drug toxicity within 3 months) as determined by medical history and/or physical exam.
- History of cancer.
- History of impaired immunoresponsiveness, including diabetes.

| | |
|---|---|
| | • Known hypersensitivity to a previous dose of influenza vaccine or allergy to eggs or any components of the study vaccines.<br>• Received influenza vaccination during the 2008-2009 season<br>• History of systemic hypersensitivity reactions to egg proteins, or any other component of FLUVIRIN®, or life-threatening reactions to previous influenza vaccinations.<br>• Has known history of Guillain-Barré Syndrome<br>• Presently receiving or history of receiving any medications or treatments that affects the immune system such as allergy shots, immune globulin, interferon, immunomodulators, cytotoxic drugs or drugs known to be frequently associated with significant major organ toxicity, or systemic corticosteroids (oral or injectable) in the past 6 months. Inhaled and topical corticosteroids will be allowed.<br>• Participated in a clinical trial or received or planned administration of an investigational compound within 30 days before receiving study vaccine and/or during the study through the Day 28 evaluation.<br>• Was vaccinated with a registered vaccine within 14 days (for inactivated vaccines) or 28 days (for live vaccines) prior to receiving the study vaccine.<br>• History of anaphylactic type reaction to injected vaccines.<br>• History of drug or chemical abuse in the year before the study.<br>• Use of new prescription medications started within 7 days before study entry.<br>• Receipt of any blood products, including immunoglobulin, within 6 months before administration of study vaccine or anticipated receipt during the study period.<br>• Donation of blood or blood products within 8 weeks before study entry or at any time during the study.<br>• Has clinical signs of active infection and/or oral temperature of $\geq$ 38 (100.4 °F). Study entry may be deferred for such individuals at the discretion of the investigator.<br>• Has evidence or history of (within the previous 12 months) drug or alcohol abuse.<br>• Any condition that, in the opinion of the investigator, might interfere with study objectives. |
| **STUDY SCHEDULE** | 1. Screening evaluations conducted to establish eligibility will be performed no more than 30 days prior to vaccination Day 0.<br>2. Vaccine administrations will occur on Day 0.<br>3. In-clinic safety evaluations will be performed at screening, before study vaccination dose (Day 0), 30 minutes after vaccination, and on Days 1, 14 and 28.<br>4. Completion of a Memory Aid for seven days following vaccine dose will be requested of subjects for use in accurate recall of local and systemic reactions. |

| | |
|---|---|
| | 5. On day of vaccination, study subjects will remain in clinic for observation for a minimum of 30 minutes after each vaccination or longer as directed by study physician.<br>6. Specimens for clinical chemistry and hematology will be collected at screening and Day 1. CRP will be collected before vaccination on Day 0 and Day 1.<br>7. Specimens for M2e antibody will be collected before vaccination on Day 0 and on Days 14 and 28.<br>8. Specimen for hemagglutinin inhibition (HAI assay) will be collected before vaccination on Day 0 and on Days 14 and 28.<br>9. Final visit on Day 28 to assess occurrence of serious adverse events, clinically severe AEs or new onset of chronic medical conditions, especially autoimmune.<br>**A detailed schematic appears in Appendix C.** |
| **SAFETY ASSESSMENTS** | • Visual assessment of the injection site on day of administration (pre-dose) and at 30 minutes post-dose and on Days 1, 14 and 28.<br>• On day of vaccination, observe study subjects in clinic for a minimum of 30 minutes post vaccination or longer as directed by the study physician or designate.<br>• Solicitation of local and systemic reactogenicity events will be captured via clinic visits using standardized grading. Reactogenicity assessment will also include unsolicited complaints.<br>• All local and systemic reactogenicity reporting in the 7 days after vaccination will be supported with the use of a Memory Aid. Clinic visit on Days 1, 14, and 28 to assess adverse events and clinical labs. |
| **IMMUNOGENICITY ASSESSMENTS** | Serum IgG responses to M2e and HAI will be assessed before vaccination on Day 0 and on Days 14 and 28. |
| **HALTING RULES** | 1. If one or more subjects experience a clinically significant AE or laboratory abnormality (Grade 3 or 4), there will be no further enrollment until a full safety review is performed.<br>2. The study will be halted (no new enrollments and no further investigational product administered) if one of the following occurs:<br>a. One subject experiences a serious adverse event (SAE) assessed as possible, probably or definitely related to investigational product or<br>b. There is a subject death assessed as possibly, probably or definitely related to investigational product.<br>A safety monitoring committee (SMC) comprised of a Principal Investigator from each site, VaxInnate, and an Independent Safety Monitor, will be set-up to assess in a blinded fashion the safety and reactogenicity should one of the above halting rules be met. The Independent Safety Monitor may be unblinded to study treatment, as needed, to adequately assess safety issues. |

| | |
|---|---|
| **FOLLOW-UP DURATION** | Volunteers will be followed for 28 days after the Day 0 vaccination. |
| **ENDPOINT PARAMETERS** | a) Safety analysis includes:<br>• Standard descriptive demography and reactogenicity from the memory aid will be assessed. All subjects who receive any investigational product will be included in the safety analyses.<br>• Subjects with adverse events (including clinical laboratory abnormalities) will be summarized by MedDRA body organ system and preferred term, severity, relatedness and, separately, by seriousness.<br>b) Immunogenicity parameters include:<br>• Geometric mean of pre- and post-vaccination anti-M2e or HAI serum antibody concentrations.<br>• Change from pre- to post-vaccination geometric mean of anti-M2e or HAI serum antibody concentrations.<br>• Proportion of subjects with an M2e and HAI-specific serum antibody titer before vaccination.<br>• Proportion of subjects with an M2e and HAI-specific serum antibody titer after vaccination.<br>• Descriptive analysis of HAI titers in subjects who received TIV plus VAX102 compared to TIV plus Placebo. |
| **DATA ANALYSIS** | Prior to analysis of primary outcome measures of safety and immunogenicity, integrity of study conduct and route of administration group comparability will be examined. M2e or HAI specific IgG antibody titration curves will be established at each time point. At the start of the study, volunteers will be scored as positive (IgG titers above an established negative baseline) or negative (IgG titers below the negative baseline) for pre-existing M2e or HAI specific IgG titers. The HAI GMT and the proportion of sero-negative volunteers who develop a four-fold or greater increase antibody titers after immunization in each group (A and B) will be determined. The HAI GMT and proportion of sero-positive volunteers who develop a significant rise in antibody titers after immunization in each group will also be estimated. Standard statistical analyses will be used to distinguish significant changes in antibody titers. One-sided 95% lower confidence bound on the estimated proportion will also be constructed. All analyses will be based upon a per-protocol cohort with additional analysis performed for the intent-to-treat (ITT) cohort. |

# 1 INTRODUCTION

Influenza, a highly communicable acute respiratory disease, is one of the major infectious disease threats to the human population. The currently licensed vaccines engender antibody responses to viral glycoproteins, HA and NA, which change from year to year and require the current vaccine to be annually updated. A vaccine that induced protective antibodies against viral structures of low or no variability could provide a constant level of long lasting immunity against influenza infection.

M2e is a promising candidate for a broadly protective influenza A vaccine as M2e undergoes little sequence variation and M2e-specific antibodies have been shown to display significant protective activity in animal models [Treanor et al. 1990]. M2e-specific antibody titers are very low or undetectable in human sera, suggesting that recovery from natural infection and current vaccines fail to induce significant M2e-specific antibody responses. Thus, humans are currently without significant M2e-specific antibody-mediated protection.

The influenza A virus ion channel, M2, is a non-glycosylated transmembrane protein that is expressed at high density in the cell membrane of viral infected cells and at low density in the lipid membrane of the mature influenza virus. Vaccines inducing antibodies to M2e will not necessarily confer protection against infection per se, but should confer protection against the development of significant clinical symptoms and disease.

VaxInnate has developed a cross-protective influenza A vaccine, VAX102, which is based on a recombinant protein expressed in *E. coli*. The protein comprises *Salmonella typhimurium* flagellin type 2, or fljB, (STF2; TLR5 ligand) fused to four tandem repeats of M2e at the C-terminus of flagellin.

**<u>Clinical studies</u>**

Three clinical studies (VAX102-01, VAX102-02 and VAX102-03) have been completed since the IND became effective in June 2007.

***VAX102-01 Summary***

This first-in-human study was a phase I multicenter, double-blind, randomized, placebo-controlled trial to assess four vaccine doses, 0.3 µg, 1.0 µg, 3 µg and 10 µg compared to placebo. Vaccine/placebo was given intramuscularly (IM) at 0 and 28 days. Clinical and laboratory safety assessments took place 1 and 7 days after immunization. Immune response to M2e and flagellin was assessed by ELISA at 7, 14 and 28 days after each dose. Immune response to M2e was measured by ELISA. Seroconversion was defined as a serum IgG anti-M2e antibody (ug/ml) value ≥ 0.0174 and a four-fold rise in titer.

The 0.3 and 1.0 ug doses were safe and well tolerated in all subjects and immunogenic in 18 (75%) of 24 vaccinees after the first dose and 23 (96%) after the second dose. In the 1.0 µg group, the geometric mean M2e antibody concentration was 0.4 µg/ml after the first dose and 1.7 µg/ml after the booster dose. Immune responses to flagellin were also robust and did not appear to negatively affect M2e antibody responses from the booster

dose. Higher doses (3 and 10 µg) showed a similar immunogenicity profile, but following the first dose, were associated with symptoms of cytokine release manifested by the presence of flu-like symptoms and elevated levels of C-reactive protein.

VAX102 was safe and able to induce high antibody levels to M2e at 0.3 and 1.0 µg doses. VAX102 is a promising new candidate M2e vaccine aimed to prevent or attenuate influenza A disease.

### *VAX102-02 summary*

The results from VAX102-01 indicated that the vaccine (VAX102) was immunogenic at the lowest dose tested. VAX102-02 was designed to test doses of 0.03 and 0.1 µg by the IM and ID routes of administration (**Table 1**). This study was performed at a single site.

**Table 1. Study design for VAX102-02**

| | Route/Volume | | |
|---|---|---|---|
| **Dose (µg)** | **IM 0.5** | **ID 0.1** | **No. of subjects** |
| **0.03** | 8 | 8 | 16 |
| **0.1** | 8 | 8 | 16 |
| **Total** | 16 | 16 | 32 |

VAX102 was well tolerated by both routes of administration. **Table 2** describes the symptoms after the first dose. The symptoms after the second dose were notably milder than the first dose (data not shown).

**Table 2** includes the 16 subjects who received their dose by IM injection. However, the ID route of administration was also well tolerated. These studies indicated that all doses of 1 µg or below could be given safely.

**Table 2. Local and systemic symptoms after the first dose of VAX 102-01/102-02 or placebo**

| **Dose** | **Placebo n=16** | **0.03 µg n=8** | **0.1 µg n=8** | **0.3 µg N=6** | **1 µg n=18** | **3 µg n=6** | **10 µg n=14** |
|---|---|---|---|---|---|---|---|
| Local [1] | | | | | | | |
| None | 11 (69) | 2 (25) | 2 (25) | 1 (17) | 3 (17) | 0 | 0 |
| Mild | 5 (31) | 3 (37.5) | 3 (37.5) | 2 (33) | 10 (56) | 3 (50) | 5 (36) |
| Moderate | 0 | 3 (37.5) | 3 (37.5) | 3 (50) | 5 (28) | 3 (50) | 8 (57) |
| Severe | 0 | 0 | 0 | 0 | 0 | 0 | 1 (7) |
| Systemic [2] | | | | | | | |
| None | 9 (56) | 6 (75) | 4 (50) | 4 (67) | 6 (33) | 2 (33) | 0 |
| Mild | 4 (25) | 2 (25) | 4 (50) | 2 (33) | 9 (50) | 0 | 2 (14) |
| Moderate | 2 (13) | 0 | 0 | 0 | 3 (17) | 4 (67) | 6 (43) |
| Severe | 1 (6) | 0 | 0 | 0 | 0 | 0 | 6 (43) |

[1] Local: injection site pain, redness, bruising or induration
[2] Systemic: headache, fatigue, joint pain, muscle aches, chills and sweats

The M2e immune response showed a dose related increase. **Figure 1** shows M2e antibody curve as defined by geometric mean concentrations measured at day 42 after two doses of VAX102 given on Days 0 and 28. At the highest doses (3 and 10 µg) the immune response plateaued at about 3 µg /ml. At a dose of 1.0 µg the antibody

concentration was 1.8 µg /ml.  The right panel of **Figure 1** demonstrates that at Day 180 M2e antibody are still above the baseline.

**Figure 1 . M2e antibody response after two doses of VAX102 at 0 and 28 days**

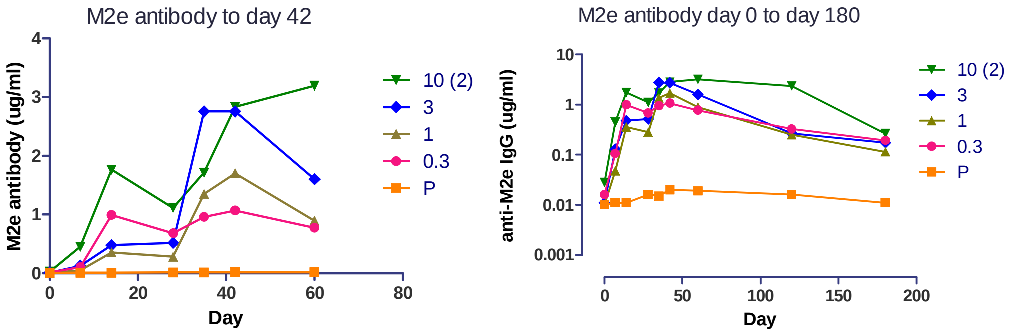


***VAX102-03 summary***

VAX102-03 was designed to study the safety and immunogenicity of VAX102 given by different routes of administration (**Table 3**).

| **Table 3. Number of volunteers listed by route of administration and dose of VAX102** | | | | |
|---|---|---|---|---|
| **Route** | **IM** | **SC** | **ID** | **No. of subjects** |
| **Volume (ml)** | **0.5** | **0.5** | **0.1** | |
| **Dose (µg)** | | | | |
| **0.3** | 8 | 8 | 8 | 24 |
| **1** | 8 | 8 | 8 | 24 |
| **2** | 8 | 8 | 0 | 16 |
| **Total** | 24 | 24 | 16 | 64 |

The study was performed at two clinical sites that had been involved in VAX102-01 and VAX102-02.  In this study, we compared the safety and immunogenicity of VAX102 given by intramuscular (IM), subcutaneous (SC) and intradermal (ID) routes of administration. Healthy volunteers were randomized to receive 0.3 µg or 1.0 µg of VAX102 either IM, SC, or ID, or 2.0 µg of VAX102 IM or SC in an open label study.

Vaccine doses were administered at Days 0 and 28. There was an assessment of symptoms at each dose level before escalating the dose. C reactive protein (CRP) assays were performed on Day 0 and 1. Serum IgG anti-M2e antibody concentration (µg/ml) was assessed by ELISA on Day 42. VAX102 was administered to 64 subjects. VAX102 was well tolerated by all subjects in the 0.3 and 1.0 µg doses. After the first dose at 2 µg, 2 subjects in the 2 µg IM group had systemic symptoms compared to none in the SC group. The mean CRP level, range and fold increase were also significantly higher in the 2 µg IM group compared to the SC group. The differences in antibody titer between the IM and SC groups were not significant.

**Table 4. CRP results listed by route of administration and dose**

| No. of subjects | Route | Dose (µg) | GM M2e | CRP (mean) | CRP (fold) |
|---|---|---|---|---|---|
| 8 | IM | 0.3 | 0.5 | 0.8 | 1.8 |
| 8 | IM | 1 | 1.3 | 0.8 | 3.1 |
| 6 | IM | 2 | 2.3 | 1.4 | 5.9 |
| 8 | SC | 0.3 | 0.7 | 0.4 | 1.8 |
| 8 | SC | 1 | 0.8 | 0.6 | 2.1 |
| 8 | SC | 2 | 1.9 | 0.8 | 3.2 |
| 8 | ID | 0.3 | 0.4 | 0.4 | 1.3 |
| 8 | ID | 1 | 1.8 | 0.7 | 2 |

## Summary of Preclinical data supporting HAI enhancement with mice

In 2007 Flulaval was tested in combination with STF2 or VAX102 in three different experiments. The H1 HAI was measured against New Caledonia (**Table 5**). In all three experiments the combination of TIV with VAX102 demonstrated a three to five fold increase in HAI titer compared to Flulaval alone.

**Table 5. Enhancement of H1 HAI with concomitant administration of VAX102 with Flulaval in mice**

| Study & report date | **1** | **2** | **3** |
|---|---|---|---|
| | **Sept '07** | **Oct '07** | **Dec '07** |
| **Flulaval** | 130 | 61 | 95 |
| **Flulaval + STF2** | 299 | 127 | - |
| **Flulaval + VAX102** | 686 | 175 | 428 |
| **Flulaval + VAX102 + alum** | - | - | 538 |
| **Flulaval to Flulaval + VAX102 fold increase** | 5.3 | 2.9 | 4.5 |

Note: 15-20 mice in each group

A similar enhancement was demonstrated for the Sanofi H5 pandemic vaccine. In this experiment (Figure 2), the combination of Sanofi H5 given with VAX102 given in the same limb showed a marked increase in HAI H5 (VN) titer.

**Figure 2. Enhancement of H5 HAI response with concomitant administration of VAX102**

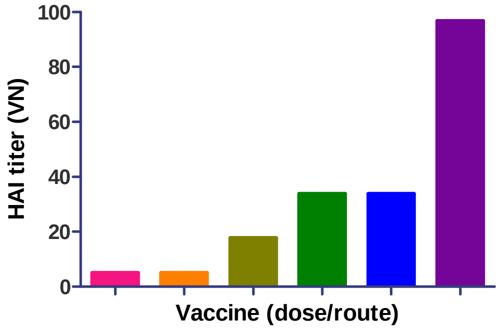


| | | | | | | |
|---|---|---|---|---|---|---|
| No. of mice | 9 | 10 | 10 | 10 | 10 | 10 |
| Sanofi H5 (VN) dose (ug) | | | 3 | | 3 | 3 |
| R3.2xHA1-2 (VN) | | | | 3 | | |
| VAX102 dose (ug) | | 3 | | | 1 | 1 |
| Injection sites | | | | | 2 | 1 |

### Rationale for current study

VAX102 is immunogenic in healthy adults. An average 6-fold increase in the H1 HAI GMT is a good indication of efficacy for the H1 component of a recombinant trivalent vaccine (Table 5). We also know that the flagellin component is able to activate the immune system via the innate immune pathway. In animal studies we have shown that this immune activation can enhance the immune response to inactivated seasonal and pandemic influenza vaccines. The immunopotentiation of the HAI immune response could be important clinically in groups like the elderly who respond poorly to TIV. The purpose of this study is to test the safety and immunogenicity of this novel adjuvant-antigen in combination with trivalent inactivated influenza vaccine in young healthy adults 18-49 years of age to determine if this combination is safe and able to adjuvant the TIV immune response. If successful, the plan is to test the combination in the elderly in a subsequent study.

## 2 OBJECTIVES

### Primary Objective

To assess the safety, reactogenicity, and tolerability of VAX102 when given with Trivalent Inactivated Influenza Vaccine (TIV) delivered in the same arm as two separate IM injections in healthy adults 18 to 49 years.

### Secondary Objectives

- To assess the immunogenicity of the VAX102 vaccine when given with TIV.
- To assess the antibody response to TIV when given with VAX102 compared to TIV alone.

# 3 STUDY DESIGN

A multicenter, double-blind, randomized, placebo controlled vaccine study to assess the safety, reactogenicity and immunogenicity of one subject group receiving 1 µg VAX102 investigational vaccine with TIV, delivered intramuscularly into the nondominant arm as a prime vaccination, compared to placebo with TIV, in healthy normal adults of both genders. All subjects will receive TIV first followed by VAX102 or Placebo and undergo identical study procedures, as outlined in **Section 6.**

Eligible subjects will be assigned to a group according to a randomization code provided. The randomization is 1:1 for each of the 2 different groups and summarized as follows:

**Table 6. Number of volunteers listed by dose and investigational vaccine**

| Groups | Vaccine 1 | Vaccine 2 | Total # Subjects |
|---|---|---|---|
| **A** | TIV (std dose) | VAX102 1 µg | 40 |
| **B** | TIV (std dose) | Placebo | 40 |
| **Total** | | | 80 |

The investigators will make a reasonable attempt to enroll an equal number of males and females into each of the groups of the study.

# 4 STUDY POPULATION

## 4.1 Number of Subjects

Eighty (80) healthy adult subjects will be recruited for enrollment at two study centers into one of two groups.

Group A is comprised 40 subjects receiving a standard dose of TIV IM first followed by 1 µg VAX102 IM.

Group B is comprised 40 subjects receiving a standard dose of TIV IM first followed by F105 (Placebo) IM.

The investigators will make a reasonable attempt to screen enough subjects to meet the enrollment for each group. Once the subject has received study vaccination on Day 0, subjects will not be replaced.

## 4.2 Inclusion Criteria

Subjects will be considered eligible to enter the study and receive vaccination if they satisfy all of the following inclusion criteria prior to receiving each vaccine dose:

- Male or female aged 18 – 49 years inclusive

- Give written informed consent to participate in the study and adherence to all protocol requirements.
- Healthy, as determined by medical history, physical examination, vital signs, and clinical safety laboratory examinations
- Females should fulfill one of the following criteria:
    1. At least one year post-menopausal,
    2. Surgically sterile,
    3. Will use oral, implantable, transdermal or injectable contraceptives for 30 days prior to first vaccination and for the duration of the study
    4. Willing to use a reliable form of contraception approved by the Investigator (*e.g.*, intrauterine device (IUD), female condom, diaphragm with spermicide, cervical cap, use of condom by the sexual partner or a sterile sexual partner) for the duration of the study.
- Women of childbearing potential must have a negative urine pregnancy test within 24 hours preceding receipt of first and booster vaccinations
- Comprehension of the study requirements, expressed availability for the required study period, and ability to attend scheduled visits.
- Did not receive influenza vaccination (TIV) during the 2008-2009 influenza season.

## 4.3 Exclusion Criteria

Subjects will be considered ineligible to enter the study and receive vaccination if they meet any of the following exclusion criteria prior to receiving each vaccination.

- Presence of significant acute or chronic, uncontrolled medical or psychiatric illness (institution of new medical or surgical treatment, or a significant dose alteration for uncontrolled symptoms or drug toxicity within 3 months) as determined by medical history and/or physical exam.
- History of cancer.
- History of impaired immunoresponsiveness, including diabetes.
- Known hypersensitivity to a previous dose of influenza vaccine or allergy to eggs or any components of the study vaccines.
- Received influenza vaccination (TIV) during the 2008-2009 influenza season.
- Has known history of Guillain-Barré Syndrome
- History of systemic hypersensitivity reactions to egg proteins, or any other component of FLUVIRIN®, or life-threatening reactions to previous influenza vaccinations.
- Presently receiving or history of receiving any medications or treatments that affects the immune system such as allergy shots, immune globulin, interferon, immunomodulators, cytotoxic drugs or drugs known to be frequently associated with significant major organ toxicity, or systemic corticosteroids (oral or injectable) in the past 6 months. Inhaled and topical corticosteroids will be allowed.
- Participated in a clinical trial or received or planned administration of an investigational compound within 30 days before receiving study vaccine and/or during the study through the Day 28 evaluation.

- Was vaccinated with a registered vaccine within 14 days (for inactivated vaccines) or 28 days (for live vaccines) prior to receiving the study vaccine.
- History of anaphylactic type reaction to injected vaccines.
- History of drug or chemical abuse in the year before the study.
- Use of new prescription medications started within 7 days before study entry.
- Receipt of any blood products, including immunoglobulin, within 6 months before administration of study vaccine or anticipated receipt during the study period.
- Donation of blood or blood products within 8 weeks before study entry or at any time during the study.
- Has clinical signs of active infection and/or oral temperature of ≥ 38 ºC (100.4 ºF). Study entry may be deferred for such individuals at the discretion of the investigator.
- Has evidence or history of (within the previous 12 months) drug or alcohol abuse.
- Any condition that, in the opinion of the investigator, might interfere with study objectives.

### 4.4 Subject Enrollment and Randomization

Eligible subjects will be assigned to a group according to a randomization code provided by an independent statistician. The independent statistician will provide the randomization information to the investigational pharmacist or designee. Within each dose group, subjects will be randomly assigned to treatment as described previously in **Table 6**.

On Study Day 0, the investigational pharmacist or designee will be provided with each eligible and consenting initials, subject ID number (SID), age and gender by the study site's personnel. The investigational pharmacist or designee will use the randomization list provided by the independent statistician to assign the next available randomization code and determine treatment assignment. For each subject randomized, the pharmacist will prepare a duplicate label bearing the subject's SID number, subject's initials, and date and affix the label to a syringe. The randomization code will be transcribed onto the Investigational Product Accountability Log along with the SID number. The investigational pharmacist or designee will then prepare the appropriate test article for each subject.

Investigational vaccine will be prepared by an unblinded investigational pharmacist who will not be involved in vaccine administration or subsequent clinical assessments.

The study design incorporates ongoing review of the safety and reactogenicity of the vaccine on Day 7 after vaccination.

Sponsor personnel involved in generation and recording of immunogenicity assays will be unblinded only after all assays and repeats are complete and final data sets are provided to data management personnel and locked in the database.

## 4.5 Unblinding

This is a phase II, double-blinded, randomized, placebo-controlled study to investigate the safety and immunogenicity of TIV plus one VAX102 dose (1.0 µg) versus TIV plus placebo. In order to allow unbiased accrual, the randomization list will be withheld from all sponsor personnel until the final subjects' data at the end of the Day 28 (± 2) assessments have been entered, after resolution of all data queries, after all immunogenicity samples have taken through Day 28 (± 2), and after the safety database has been locked.

Treatment assignments will be provided:

- to sponsor personnel not involved in the performance of immunogenicity assays 28 days after the investigational vaccine dose (and completion of safety data) as above,
- to clinical site personnel only after all case report forms are verified, all queries are resolved, and the entire safety database locked,
- to sponsor personnel involved in the generation and recording of immunogenicity data only after all assays and repeats are complete and final datasets are provided to data management personnel and locked in the database.

## 4.6 Withdrawal of Subjects from the Study

Every reasonable effort should be made to ensure that each subject complies with the protocol and completes all study visits. However, a subject may withdraw - or be withdrawn involuntarily - from participation if:

- The subject withdraws consent.
- The investigator recommends discontinuation in the interest of the subject's safety (i.e., adverse event related) or because of significant and irremediable protocol non-compliance.
- The subject becomes pregnant as evidenced by a positive pregnancy test.
- VaxInnate, the IRB, or the FDA terminates the clinical trial for safety or other reasons.

Subjects may be withdrawn from treatment and, if consenting, continue to complete a per-protocol safety evaluation. Where this is deemed impractical (e.g., a chronically non-compliant subject) or where the subject does not consent to further follow-up, a complete Day 28 (± 2) **(Section 6.2.4**) and study closeout visit case report form(s) will be completed on any subject prematurely withdrawn from the clinical trial on the day of withdrawal. Any adverse event leading to early discontinuation will be followed to resolution. Any woman who becomes pregnant during the trial will be followed until delivery, and the outcome of the pregnancy documented. No subject prematurely withdrawing/withdrawn will be replaced.

### 4.7 Halting Rules

- If one or more subjects experience a clinically significant AE or laboratory abnormality (Grade 3 or 4), there will be no further enrollment until a full safety review is performed.
- The study will be halted (no new enrollments and no further investigational product administered) if one of the following occurs:
  a. One subject experiences a serious adverse event (SAE) assessed as possible, probably or definitely related to investigational product or
  b. There is a subject death assessed as possibly, probably or definitely related to investigational product.

A safety monitoring committee (SMC) comprised of a Principal Investigator from each site, VaxInnate, and an Independent Safety Monitor, will be set-up to assess in a blinded fashion the safety and reactogenicity should one of the above halting rules be met. The Independent Safety Monitor may be unblinded to study treatment, as needed, to adequately assess safety issues.

## 5 STUDY PRODUCT DESCRIPTION

### 5.1 Investigational Product

#### 5.1.1 VAX102 (STF2.4xM2e[Hu])

The VAX102 will be supplied in individual 3 mL glass vials containing 0.75 mL of vaccine at a concentration of 20 µg of STF2.4xM2e(Hu) per 1 mL. The STF2.4xM2e(Hu) vaccine is formulated in a Phase I formulation buffer (F105) that contains 10 mM Tris, 10 mM Histidine, 5% (w/v) sucrose, 75 mM NaCl, 0.1 mM EDTA, 0.5% (v/v) ethanol and 0.02% (w/v) polysorbate-80 at pH 7.2.

The study design incorporates one dose level 1 µg. This 1 µg dose level will be prepared by a field mix procedure where a specific amount of vaccine is combined with a specified volume of VAX102 vaccine diluent (F105). The investigational pharmacist or trained study personnel compounding the individual subject dosages will follow a dose specific procedure to ensure the proper final dosage strength is achieved. This compounding step will be performed on the day of administration (Day 0); the individual vials for the each subject will be provided along with the compounding procedure.

#### 5.1.2 Trivalent Inactivated Influenza Vaccine (TIV)

**FLUVIRIN®** (Novartis) Expiration date: 30 June 2009

Each 0.5-mL dose contains a total of 45 µg of influenza virus hemagglutinin (HA) from each of the following 3 strains: A/Brisbane/59/2007(H1N1); A/Uruguay/716/2007 (H3N2), an A/Brisbane/10/2007-like strain; and B/Florida/4/2006.

#### 5.1.3 Placebo (F105 – Formulation Buffer)

F105 buffer contains 10 mM Tris, 10 mM Histidine, 5% (w/v) sucrose, 75 mM NaCl, 0.1 mM EDTA, 0.5% (v/v) ethanol and 0.02% (w/v) polysorbate-80 at pH 7.2. It is the buffer that is used to formulate VAX102.

### 5.2 Storage and Handling

Individual vials of VAX102 vaccine and diluent (F105) will be provided by VaxInnate Corp. The VAX102 vials and diluent should be stored at a controlled temperature (≤-60ºC) in a secured freezer until use. On each dosing day, VAX102 vials and diluent required for vaccinating the subjects should be removed from the freezer and placed at room temperature until visibly thawed (~ 15 minutes) prior to dosing or diluting by the investigational pharmacist or trained study personnel. The vaccine or diluent in each vial will be inspected to ensure that it is completely thawed and then gently swirled by the investigational pharmacist or trained study personnel to ensure it is thoroughly mixed prior to it being drawn into a syringe for compounding or for injection into a study subject. All vials removed from the freezer should be used within 2 hours and must NOT be re-frozen and NOT used thereafter. After each dose preparation, the pharmacist will place the used vial(s) in a kit box, seal the kit box with a label with the subject's randomization number and store at room temperature**.**

FLUVIRIN® will be supplied as 5 mL multi-dose vials to study sites and stored under manufacturer's instructions.

### 5.3 Investigational Product Preparation

On Study Day 0, the investigational pharmacist or trained study personnel will be provided with each eligible and consenting subject's initials, subject ID number (SID), and gender by the study site's personnel. The investigational pharmacist or trained study personnel will use the list to determine the Group and treatment assignment. The investigational pharmacist or trained study personnel will prepare two labels bearing the subject's SID number, initials, date, and **vaccine 1** or **vaccine 2**. The label with **vaccine 1** will be affixed to the **TIV vaccine syringe,** and the label with **vaccine 2** will be affixed to the **VAX102 vaccine syringe**. The subject's SID number and randomization number will be transcribed onto the Investigational Product Accountability Log.

The investigational pharmacist or trained study personnel will then prepare the appropriate study vaccine dose dilution for each subject.

The one clinical dose of STF2.4xM2e(Hu) (VAX102) will be achieved as described below **Section 5.3.1**. Each vial of vaccine contains 0.75 mL of vaccine at a concentration of 20 ug/mL. Each vial of vaccine diluent contains 1.8 mL.

The placebo (F105) will be achieved as described below in **Section 5.3.2.**

The 0.5 ml dose of **FLUVIRIN®** will be achieved as described below in **Section 5.3.3.**

After study vaccine preparation is completed for a given day, any opened stock vaccine vials will be restoppered and retained for review by a VaxInnate representative, sent to VaxInnate or destroyed as per site's standard operating procedure.

#### 5.3.1 VAX102 Vaccine Dilution Scheme Group A Dose 1.0 µg for 0.5 mL volume for IM Injection

For all dilutions use 0.3 mL Lo-Dose syringe

1. Remove 0.20 mL from the 20 µg/mL vaccine vial. Dispense the entire volume into the vial of F105 diluent. The final volume is 2.0 mL (0.2 + 1.8 mL) at 2.0 µg/mL.
2. Inject 0.50 mL IM of this 2.0 µg/mL solution to deliver 1.0 µg VAX102.

#### 5.3.2 Placebo (F105) Group B for 0.5 mL volume for IM Injection

*NO DILUTION NEEDED*

1. Remove 0.5 mL from the F105 diluent vial.

#### 5.3.3 TIV Vaccine for Groups A-B STD for 0.5 mL volume for IM Injection

*NO DILUTION NEEDED*

1. Remove 0.5 mL from FLUVIRIN® vial for injection of influenza virus hemagglutinin (HA) from 3 strains.

### 5.4 Investigational Product Administration

All investigational products are to be administered under the supervision of the Principal Investigator or a qualified subinvestigator designated to VaxInnate in writing prior to the trial and trained in both the protocol and contents of the Investigator's Brochure. Under no circumstances will the Principal Investigator allow investigational products to be used other than as directed by this protocol. Investigational products will be administered as follows:

Study vaccines will be prepared as described in **Section 5.3** and administered within two (2) hours of dilution.

Administration of the vaccine in all parts of the study will be <u>intramuscular injection</u> into the deltoid muscle region by **a 1 inch 23 gauge needle and 1 mL syringe on Day 0**. In all vaccinations a standard dose of TIV IM will be administered first followed by VAX102 or Placebo [F105]. Both vaccinations will be given into the <u>non-dominant arm</u> of each subject. The arm used (right or left) will be recorded in the subject's case report form (CRF). Each site should be wiped with an alcohol wipe before injection of the vaccine. The times when the subjects' vaccinations were given should be recorded in the CRF. At the time of vaccine preparation, duplicate labels will be prepared that contain the subject ID number, initials, date, and **vaccine 1** and **vaccine 2**. These labels will be attached to or accompany the filled syringes. One of the labels will be removed and attached to the subject's Case Report Form for verification of correct dose.

### 5.5 Accountability of Investigational Products

The Principal Investigator or a designee is responsible for maintaining complete investigational products inventory records accounting for receipt, storage, dispensation, and final disposition using forms supplied by (or local equivalents approved by) VaxInnate or designee. These records will be reviewed by VaxInnate representatives. At the conclusion of the trial all vials of investigational products, used and unused, will be returned to VaxInnate or destroyed as per site's SOP only after VaxInnate's approval.

## 6 STUDY SCHEDULE

The site will recruit the subjects and designated site personnel will perform all procedures according to the protocol.

### 6.1 Screening Visit (day -30 to -1)

The following procedures will be done at the indicated times (see also **Appendix C** for a Schedule of Procedures and Assessments).

- Potential subjects will be instructed in the nature of the study, the intended procedures, and risks and benefits of study participation. Questions will be solicited. Signed and dated written, informed consent will be obtained and persons not giving consent will not participate further.
- Inclusion and exclusion criteria will be reviewed to assure subject eligibility.
- Demographics and a medical history will be obtained and documented.
- A complete baseline physical examination (HEENT, heart, lungs, abdomen, skin, lymph nodes, neurological, musculoskeletal systems), height and weight including vital signs (heart rate [HR], blood pressure [BP], respiratory rate [RR], oral temperature [Temp]), will be performed and recorded at this visit.
- Subjects eligible by history will provide approximately 20 mL of venous blood and a urine sample will be used for baseline clinical laboratory tests:
    - Hematology: complete blood count (CBC) including hemoglobin (Hgb), hematocrit (Hct), white blood cell (WBC) with differential, and platelet count
    - Chemistry: serum SGPT/ALT and SGOT/AST, blood urea nitrogen (BUN), creatinine, glucose,
    - Urinalysis: glucose, protein, blood via multistix or equivalent dipstick (trace protein is acceptable),
    - In females of childbearing potential, a negative urine pregnancy test
- Subjects will be asked to provide phone contact information.

### 6.2 Dosing and Early Follow-up

#### 6.2.1 Day 0 Vaccination

***Pre-Dosing***

- An interval medical history will be performed to ensure that each subject continues to meet all inclusion and no exclusion criteria.
- Vital signs (HR, BP, RR, Temp) will be obtained and recorded.
- A targeted physical examination of axillary lymph nodes, injection site evaluation, and baseline sign and symptom assessment will be performed and recorded by qualified study personnel. Other body system examination will be performed and recorded, if directed by subject's symptoms, by qualified NP, PA, or physician.
- Approximately 10 mL of venous blood will be evaluated for serology (serum M2e and flagellin antibody tests) and Hemagglutination inhibition assay (HAI).
- Approximately 5 mL of venous blood will be evaluated for C-reactive protein (CRP).
- A urine specimen will be collected from each female subject of childbearing potential and tested in the clinic for evidence of pregnancy. No test article will be administered until a negative result is obtained and documented.

***Prime Dosing and 30-Minute (+15) Follow-up***
On day of vaccination, study subjects will remain in clinic for observation for a minimum of 30 minutes post vaccination or longer as directed by study physician or designate.

- TIV (Vaccine 1) followed by VAX102 or Placebo (Vaccine 2) will be administered IM as described in **Section 5.4.** (*Subjects who receive any amount of test article will be considered enrolled and followed through Day 28 (± 2) for safety and immunogenicity. No additional follow-up will be required of subjects who are screened but do not receive any test article*.)
- At 30 minutes (+15 minutes) after dosing, each subject will have the following:
  - vital signs (HR, BP, RR, Temp),
  - targeted assessment of axillary lymph nodes and site of injection performed by qualified study personnel and recorded. Other body system examination will be performed and recorded, if directed by subject's symptoms, by qualified study personnel. New abnormalities will be captured as adverse events and any medications taken captured on concomitant medications.
- Subject will be provided with a Memory Aid, ruler and a digital thermometer, and instructed on their use. In the clinic the subjects will take his/her temperature orally and measure the injection site area of pain and/or induration. The Memory Aid will be used from Day 0 to Day 6.
- Subjects will be instructed to return for the Day 1 clinic visit.

### 6.2.2 Day 1 (+1)

- Vital signs (HR, BP, RR, Temp) will be taken and recorded.
- Subjects will be queried regarding the occurrence of adverse events, Memory Aid reviewed (if available) and any new concomitant medications taken, and the results recorded.

- A targeted assessment of axillary lymph nodes and site of injection will be performed by qualified study personnel and recorded. Other body system examination will be performed and recorded, if directed by subject's symptoms, by qualified personnel. New abnormalities will be captured as adverse events.
- Approximately 20 mL of venous blood will be evaluated for the following laboratory tests:
  - Hematology: complete blood count (CBC) including hemoglobin (Hgb), hematocrit (Hct), white blood cell (WBC) with differential, and platelet count
  - Chemistry: serum SGPT/ALT and SGOT/AST, blood urea nitrogen (BUN), creatinine, glucose
  - C-reactive protein (CRP)
- Subjects will be instructed to return for the Day 14 (± 2) clinic visit and bring back their Memory Aid.

#### 6.2.3 Day 14 (±2)

- Vital signs (HR, BP, RR, Temp) will be taken and recorded.
- Subjects will be queried regarding the occurrence of adverse events, Memory Aid reviewed and retained, and any new concomitant medications taken, and the results recorded.
- A targeted assessment of axillary lymph nodes and site of injection will be performed by qualified study personnel and recorded. Other body system examination will be performed and recorded, if directed by subject's symptoms, by qualified personnel. New abnormalities will be captured as adverse events.
- Approximately 10 mL will be evaluated for serology (serum M2e, flagellin and HAI antibody tests).

#### 6.2.4 Day 28 (±2)

- Vital signs (HR, BP, RR, Temp) will be taken and recorded.
- Subjects will be queried regarding the occurrence of adverse events, any new concomitant medications taken, and the results recorded.
- A targeted assessment of axillary lymph nodes and site of injection will be performed by qualified study personnel and recorded. Other body system examination will be performed and recorded, if directed by subject's symptoms, by qualified personnel. New abnormalities will be captured as adverse events.
- Approximately 10 mL will be evaluated for serology (serum M2e, flagellin and HAI antibody tests).
- This contact completes subjects' formal participation in protocol VAX102-07.

#### 6.2.5 Unscheduled Visit

At anytime during the course of this study between scheduled clinic visits, the study subject will be asked to initiate a call to the study staff to report any concerning symptoms.

- This study subject will be interviewed by a clinical study team member and any subjects who report moderate (Grade 2) or greater symptoms will be considered for a visit for further evaluation.

Evaluation will include:

- Vital signs (HR, BP, RR, Temp) will be taken and recorded.
- Subjects will be queried regarding the episode of illness and any concomitant medications taken, and the results recorded as adverse event(s).
- A targeted physical examination directed by subject's symptoms will be performed. New abnormalities will be captured as adverse events.
- If symptoms as suspected by the investigator are more serious, the subject will be referred to his/her primary care physician or local emergency room for further evaluation and treatment.

*Refer to* ***Section 4.6*** *for Early Withdrawals or Discontinuations*.

# 7 STUDY PROCEDURES AND EVALUATIONS

## 7.1 Clinical Procedures and Evaluations

### 7.1.1 Screening

Subjects will be invited to give written informed consent after having the protocol described to them, review inclusion/exclusion criteria and being given the opportunity to read the consent form, and having their questions answered. During the screening visit, consenting subjects will have his/her medical history confirmed and selected clinical laboratory tests to ensure eligibility. Those subjects remaining eligible after evaluation of screening clinical laboratory values will return on Day 0 for review of eligibility and for vaccination.

### 7.1.2 Medical History and Physical Examination

A complete physical examination (HEENT, heart, lungs, abdomen, skin, lymph nodes, neurological, musculoskeletal systems) (screening visit days -30 to -1) and medical history/review of systems will be performed and recorded before the first dosing (prime).

The medical history should record significant problems active at the time of screening or within the prior year. Problems that have been clinically inactive within the prior year, but which might alter the subject's current or future medical management, should also be noted (e.g., known mitral valve prolapse, history of seizure disorder, etc.). Completely resolved past problems with no impact on current medical management (e.g., a healed fracture at age 12) may be omitted.

Vital signs (HR, BP, RR, oral Temp) will be included in the physical examination, and any significant abnormalities in the physical examination will be recorded on the case report forms.

### 7.1.3 Targeted Physical Examination, Baseline Signs and Symptoms, and Injection Site Assessment, and Vital Signs Before and/or After Vaccination

A targeted physical examination to include assessment of axillary lymph nodes, injection site evaluation, and vital signs will be obtained and recorded at Day 0 before vaccination, 30 minutes after vaccination, and on Days 1, 14 (±2), and 28 (±2), and unscheduled visits, if applicable.

Targeted physical examinations as needed for evaluation of complaints during the trial may be performed at the discretion of the investigator. These examinations may include any other body system directed by subject's symptoms and will be recorded using a standardized worksheet. Any new abnormalities will be captured as adverse events.

### 7.1.4 Safety and Tolerability Evaluation

Following vaccination, each subject will maintain a written Memory Aid of potential vaccine reactogenicity for seven (7) days after vaccination (Days 0 - 6). Subjects will be asked to return the Memory Aid at Day 1 for review if available and on Day 14 with abstraction of the data from the Memory Aid at that time.

Clinical laboratory studies will be performed at screening and at Day 1 (+1).

Ascertainment of adverse events and new or changes in concomitant medications will be carried out at each visit through early termination or study completion Day 28 (± 2).

Specific definitions and grading for vaccine reactogenicity symptoms and findings and adverse events are outlined in later sections and related appendices. Subjects will be followed until resolution of adverse events or until their clinical status has clearly stabilized and will be referred to their local physician for any unresolved issues.

### 7.1.5 Concomitant Medications

Subjects should be instructed not to introduce new medications without consulting or notifying the investigator or his designee. Subjects will be questioned regarding new medications at each visit through Day 28 (± 2) days. Any such medications will be recorded on the case report form, and also utilized by site staff as a prompt to query the subject regarding potential adverse events triggering changes in concomitant medications.

## 7.2 Laboratory Evaluations

### 7.2.1 Clinical Laboratories

Hematology: The following will be done at the screening visit and Day 1 after vaccination.

- Complete blood count (CBC) including hemoglobin (Hgb), hematocrit (Hct)
- White blood cell count (WBC) with differential,
- Platelet count

Clinical Chemistry: The following will be done at the screening visit and Day 1 after vaccination.

- SGPT/ALT and SGOT/AST
- Blood urea nitrogen (BUN)
- Creatinine
- Glucose

CRP will be done before vaccination on day 0 and Day 1 after vaccination.

Urinalysis: A urine sample will be obtained at the screening visit and if clinically indicated to evaluate illness at any other time. The following will be tested in the clinical laboratory:

- Glucose
- Protein
- Hemoglobin

Urinalysis with a multistix or equivalent dipstick is acceptable.

Pregnancy Testing: Urine β-HCG determination will be performed on a freshly-obtained urine specimen on the day of screening and on Day 0 prior to vaccination on each female subject of child bearing potential. A negative result must be obtained and recorded before any test article is administered.

### 7.2.2 Specific Antibody Assays

A venous blood sample (approximately 10 mL) will be taken from each subject pre-vaccination on Day 0 and on Days 14 and 28 for the analysis of anti-M2e serum antibody titers and HAI antibodies.
The immunogenicity of the VAX102 vaccine will be evaluated by measuring the number of subjects who demonstrate seroconversion either by developing a measurable titer following vaccination or by showing a significant increase in anti-M2e serum antibody titers post-vaccination. Standard statistical tests will be used to define a significant rise in antibody titers.

In addition to HAI titers (H1, H3 and B components), sera Days 0, 14 (±2) and 28 (±2) will also be evaluated for microneutralization titers, anti-flagellin titers using a panel of exploratory assays.

### 7.2.3 Specimen Collection, Preparation, Handling, and Shipping

The instructions for handling each type of specimen are found in the laboratory instructions or procedures.

# 8 ASSESSMENT OF SAFETY

## 8.1 Definition of Immediate Complaints, Vaccine Reactogenicity and Adverse Events

### 8.1.1 Immediate Complaints

Immediate complaints as a result of vaccination (see **Appendix A**) are recorded on the symptom assessment CRF after 30 minutes of observation. The Memory Aid given to the subject will be used to capture symptoms for the next 7 days. The Memory Aid provides uniform definitions for grading severity.

### 8.1.2 Vaccine Reactogenicity

A selection of subjective complaints (see **Appendix A)** reasonably anticipated to occur as a result of receipt of the test articles are provided in the Memory Aid. These will be solicited and recorded by the subject. A selection of potential physical findings that might occur as a result of vaccination is provided on the Targeted Physical Assessment. Findings from these two sources will be abstracted to the case report forms and reported as vaccine reactogenicity. These findings should NOT be additionally recorded as adverse events unless they fulfill the criteria set forth in **Section 8.1.5.**

Severity definitions used to grade reactogenicity events are included as part of the Targeted Physical Assessment and Memory Aid. Subjects will record at the level of greatest severity of each reaction type experienced each day during the seven-day post each vaccination interval. Counts and proportions of study vaccine recipients reporting each class of solicited reaction in the first seven days post vaccination will be summarized by severity grade, and by all non-zero severity grades.

### 8.1.3 Adverse Events

An adverse event (AE) is any unfavorable, harmful, or pathologic change in a research subject as indicated by physical signs, symptoms and/or clinically significant laboratory abnormalities that occurs in association with the use of a product (trial-related), whether or not considered to be product-related. This includes intercurrent illnesses, injuries, worsening of pre-existing conditions, and events occurring as a result of product abuse or overdose. Stable pre-existing conditions and/or elective procedures are not adverse events.

Adverse events are captured after all screening procedures are completed and subject receives the initial dose of investigational product on Day 0. All events fulfilling any part of the AE definition must be recorded on the Adverse Event CRF (with the exception of pre-defined immediate complaints or symptoms/signs of vaccine reactogenicity in the first seven (7) days following immunization, which are captured on other specific instruments; see above) and graded according to the Adverse Event and Clinical Laboratory Grading tables in **Appendix B**.

Treatments and procedures are not adverse events; rather, the illness which

precipitates them should be recorded. For example, “cholecystectomy”, is not an AE, but the diagnosis of “cholecystitis” or “gall stones” leading to the surgical (or medical) treatment is an AE. Where possible, specific diagnoses are preferred in reporting AEs. Individual complaints or findings may be reported as AEs, but when multiple complaints or findings occur together and can be logically and defensibly assembled into a single clinical syndrome or diagnosis, the latter is preferred. For example, an isolated finding of elevated AST and ALT would be recorded as “elevated transaminases.”

#### 8.1.4 Clinical Laboratory Findings as Adverse Events

Clinical laboratory measures, except for urine pregnancy tests, are not required by the protocol. It is possible that laboratory tests could be ordered to assess an adverse event. Clinical laboratory abnormalities may be adverse events in themselves, and should be reported as adverse events if they raise clinical concern or are sufficient to initiate additional diagnostic tests. Clinical laboratory abnormalities that form part of a coherent clinical syndrome (e.g., transaminase elevations in well-defined hepatitis as described in **8.1.3** above) need not be listed as separate adverse events so long as the syndrome of which they are components is captured (e.g., it is not necessary to capture “hepatitis” and “elevated hepatic transaminases” and “anorexia” separately – the first of these includes the latter two). Clinical laboratory abnormalities that are reported as adverse events should be followed to resolution or until stable, and ancillary case report forms will be provided to capture these data. Subjects with persistent abnormalities will then be referred to their local physician for any unresolved issues.

**Severity of Event:** All AEs will be assessed by the clinician using a protocol defined grading system (see **Appendix B**). For events not included in the protocol grading system, the following guidelines will be used to quantify intensity.

- Mild: events require minimal or no treatment and do not interfere with the patient’s daily activities.
- Moderate: events result in a low level of inconvenience or concern with the therapeutic measures. Moderate events may cause some interference with functioning.
- Severe: events interrupt a patient’s usual daily activity and may require systemic drug therapy or other treatment. Severe events are usually incapacitating.
- Life threatening: any adverse drug experience that places the patient or subject, in the view of the investigator, at immediate risk of death from the reaction as it occurred, i.e., it does not include a reaction that had it occurred in a more severe form, might have caused death.

Changes in the severity of an AE should be documented to allow an assessment of the duration of the event at each level of intensity to be performed. Adverse events characterized as intermittent require documentation of onset and duration of each episode.

**Relationship to Study Products:** The clinician's assessment of an AE's relationship to test article is part of the documentation process, but it is not a factor in determining what is or is not reported in the study. If there is any doubt as to whether a clinical observation is an AE, the event should be reported. All AEs must have their relationship to study product assessed. In a clinical trial, the study product must always be suspect. To help assess, the following guidelines are used.

- Unrelated: The adverse event is clearly not related to the Investigational Product(s). This category applies to those adverse events, which, after careful consideration are clearly due to extraneous causes.
- Possibly: The adverse event may be related to the Investigational Product(s). This category applies when there is modest suspicion that the experience may be related to study medication, but there is also suspicion that other etiologies (e.g. concomitant illness or medication, etc.) are also possible.
- Probably: The adverse event is likely related to the Investigational Product(s). This category applies when the experience most likely seems to be related to the study medication but there is a modest suspicion that it could be related to other causes.
- Definitely: The adverse event is clearly related to the Investigational Product(s). This category applies when after careful medical consideration there is almost no consideration of other causation.

### 8.1.5 Double Reporting of Reactogenicity Findings/Complaints

As per sections **8.1.1**, and **8.1.2** above, findings and/or complaints specifically captured by the Memory Aid should NOT, in general, be additionally recorded as AEs if they occur within seven (7) days of a study vaccine dose.

The rationale for not doubly-reporting reactogenicity events as AEs is as follows. The specified range of reactogenicity complaints and findings are those reasonably expected to be associated with IM influenza vaccine. These data are collected by clinical study team member(s) and transferred to CRFs, analyzed, and reported in detail to investigators and regulatory authorities as *vaccine reactions* and are *presumed to be product-related*. Separation of these numerically frequent, but usually minor, vaccine reactions from other classes of adverse events has two benefits to the safety analysis. It markedly simplifies the careful analysis of the frequency and duration of these reactions and their comparison among treatment groups. In addition, these reports would, if reported as routine AEs, tend to cluster in a few MedDRA body systems. Separating these common, expected vaccine reactions from other AEs allows for the elucidation of other potentially important, but less common, types of events in the statistical analysis of AEs in those body systems.

There are *three exceptions* to the rule regarding double reporting:

a) Any of these findings or complaints which fulfills the definition of "Serious Adverse Event" (see **Section 8.2**) should be recorded as an AE (and reported as per **Section 8.3**),

b) Any of these findings or complaints that persists beyond seven (7) days after a study vaccine dose should be recorded as an AE,

c) Any of these findings or complaints which the investigator *unequivocally* categorizes, based on strong clinical evidence, as unrelated to the test article should be recorded as an AE. In this case the investigator *must* assign a causality of "unrelated" (not "unknown") and record a clear rationale in source documents.

If a reactogenicity finding or complaint is doubly-recorded as an AE, site personnel should take care to record a verbatim AE identical to the wording of the reactogenicity finding/complaint, and also an AE start date identical to the onset date implied by the reactogenicity case report forms. The sponsor will then report such events as both reactogenicity and AE findings, but will be able to link the two reports in the database for discussion and analysis purposes.

## 8.2 Definition of Serious Adverse Events

A serious adverse event (SAE) is any adverse event that results in any of the following outcomes:

a) death,

b) an immediate threat to life,

c) inpatient hospitalization or prolongation of an existing hospitalization,

d) persistent or significant disability/incapacity, or

e) a congenital anomaly/birth defect.

Important medical events that do not result in one of these outcomes, but, based on appropriate medical judgment, are deemed to jeopardize the subject or require medical or surgical intervention to avert one of the listed outcomes, may also be considered SAEs at the investigator's discretion.

## 8.3 SAE Reporting Procedures

### 8.3.1 SAE Detection and Reporting

Any AE considered serious by the PI or Subinvestigator or which meets the aforementioned criteria must be submitted on an SAE form to Vaxinnate Corporation by to the email or fax listed below. Questions about SAE reporting can also be referred to Dr. Taylor using the same contact information.

Dr. David Taylor
Email: david.taylor@vaxinnate.com or his designee
Virginia Suppers Email: Virginia.suppers@vaxinnate.com

VaxInnate Corporation
3 Cedar Brook Drive
Cranbury, NJ 08512
Tel: 609-860-2289 cellular 919-349-6109 or Fax: 609-860-0022
V. Suppers 609-860-2857

The study clinician will complete a Serious Adverse Event Form within the following timelines:

- All deaths and immediately life-threatening events, whether related or unrelated, will be recorded on the Serious Adverse Event Form and sent by fax within 24 hours of site awareness.
- Serious adverse events other than death and immediately life-threatening events, regardless of relationship, will be reported via fax by the site within 72 hours of becoming aware of the event.

All SAEs will be followed until satisfactory resolution.

All SAEs must also be reported by the PI as soon as possible to the institutional review board reviewing and approving the clinical trial. It is the responsibility of VaxInnate to report the event as specified in 21 CFR Part 312.32: fatal and life-threatening events within 7 calendar days (by phone or fax) and all other SAEs in writing within 15 calendar days. All serious events designed as “unrelated” to study product(s), will be reported to the FDA at least annually in a summary format.

### 8.3.2 Reporting of Pregnancy

Any finding of a positive pregnancy test or notification of the same should be reported to the VaxInnate Study Physician within 72 hours of notification:

Dr. David Taylor
Email: david.taylor@vaxinnate.com or his designee
Virginia Suppers Email: Virginia.suppers@vaxinnate.com

VaxInnate Corporation
3 Cedar Brook Drive
Cranbury, NJ 08512
Tel: 609-860-2289 cellular 919-349-6109 or Fax: 609-860-0022
V. Suppers 609-860-2857

Pregnancy is not considered an adverse event, but is reportable to the IRB and CBER in the annual report. Administration of further doses of vaccine should cease immediately, although the subject should be followed for the usual safety

evaluations and blood draws. The pregnancy progress, state of prenatal care, and safety and outcome of mother and child (if the pregnancy is taken to delivery) should be followed and reported to the Sponsor.

### 8.3.3 Procedures to be Followed in the Event of Abnormal Laboratory Test Values or Abnormal Clinical Findings

A grading scale for abnormalities of clinical laboratory measures mandated by the protocol is provided as **Appendix B**. Isolated clinical laboratory abnormalities may be adverse events in themselves, and should be reported as adverse events if they raise clinical concern or are sufficient to initiate additional diagnostic tests. Clinical laboratory abnormalities that form part of a coherent clinical syndrome (e.g., transaminase elevations in well-defined hepatitis as described in **8.1.3** above) need not be listed as separate adverse events so long as the syndrome of which they are components is captured (e.g., it is not necessary to capture “hepatitis” and “elevated hepatic transaminases” and “anorexia” separately – the first of these includes the latter two). Clinical laboratory abnormalities that are reported as adverse events should be followed to resolution, and ancillary case report forms will be provided to capture these data.

### 8.3.4 Type and Duration of Follow-up of Subjects after Adverse Events

All AEs should be followed to resolution. AEs persisting to the final follow-up should be followed until the symptoms resolve or stabilize.  Then the subject will be referred to their local physician.

## 8.4 Halting Rules

- If one or more subjects experience a clinically significant AE or laboratory abnormality (Grade 3 or 4), there will be no further enrollment until a full safety review is performed.
- The study will be halted (no new enrollments and no further investigational product administered) if one of the following occurs:
  c. One subject experiences a serious adverse event (SAE) assessed as possible, probably or definitely related to investigational product or
  d. There is a subject death assessed as possibly, probably or definitely related to investigational product.

## 8.5 Safety Oversight

VaxInnate will serve as the Sponsor for this study.  Sponsor responsibilities will include the creation and oversight of the protocol development, submission of the Investigational New Drug (IND) application to the Food and Drug Administration (FDA), monitoring and ensuring Good Clinical Practice (GCP) conduct of the study, and the submission of annual and final study reports to the FDA.

If there appears to be a safety issue which would cause the halting of the trial, the site will alert VaxInnate and inform them of their findings.

A safety monitoring committee (SMC) comprised of a Principal Investigator from each site, VaxInnate, and an Independent Safety Monitor, will be set-up to assess in a blinded fashion the safety and reactogenicity should one of the above halting rules be met. The Independent Safety Monitor may be unblinded to study treatment, as needed, to adequately assess safety issues.

If the trial is halted or amendments are recommended, the sites' IRBs, and FDA will be notified.

# 9 DATA COLLECTION, PROCESSING AND ANALYSIS

## 9.1 Data collection and processing

Subject screening/enrollment will be documented using an electronic master subject log. This log will capture the following information: subject number, initials, date screen/enroll and reason for not enrolling.

In this study, CRFs utilizing Smart Pen™, a digital pen technology, will be used to gather originally-captured data. Therefore, the CRFs will serve as the source document for all protocol procedures and assessments, except for concomitant medications, adverse events, and all "Symptom Assessments" obtained from subject-completed memory aids (MEMA CRF). For these required procedures and assessments, the collected data will be recorded onto logs or memory aids as source documentation. This data will then be transcribed onto the CRF by the study staff. An electronic data management system will be used to create, modify, maintain, archive, retrieve, and transmit study data.

The Investigator remains responsible for the accuracy and adequacy of all data entered on the CRFs. Data will be monitored as described in **Sections 10.7 and 10.8**. Under direction of the clinical monitor, CRFs will be source data verified, as appropriate and further processed. Upon further data processing, queries may be generated and sent to the Investigator for clarification or correction. The Investigator will address any queries and resolutions verified by the clinical monitor.

## 9.2 Statistical methods

### 9.2.1 Overview and Study Objectives

A multicenter, double-blind, randomized, placebo controlled vaccine study to assess the safety, reactogenicity and immunogenicity of two groups receiving TIV with 1 µg VAX102 investigational vaccine, delivered intramuscularly as a prime vaccination, compared to TIV with placebo, in healthy normal adults of both genders.

The objectives of the study are:

**Primary:** To assess the safety, reactogenicity, and tolerability of VAX102 when given with Trivalent Inactivated Influenza Vaccine (TIV) delivered in the same arm as two separate IM injections in healthy adults 18 to 49 years.

**Secondary:**

- To assess the immunogenicity of the VAX102 vaccine when given with TIV.
- To assess the antibody response to TIV when given with VAX102 compared to TIV alone.

### 9.2.2 Sample size and power

Selection of sample size is arbitrary, and chosen to be appropriate to the phase II nature of the trial.

### 9.2.3 Analysis populations

- The safety population will consist of all subjects who received at least one immunization of investigational product (vaccine).
- The primary immunogenicity population will consist of all subjects who receive both immunizations of study treatment (VAX102 or placebo and TIV) and have baseline and Days 14 and 28 (± 2) anti-M2e or HAI serum antibody titers, and who have no major protocol violations that could compromise the interpretation of immunology results.

### 9.2.4 Study Hypothesis

The hypothesis is that the VAX102 vaccine with TIV, administered IM, will be generally well-tolerated and will elicit M2e or HAI antibodies respectively in at least one dose level tested, that are comparable to, or greater than, those elicited by the placebo (F105) with TIV delivered IM.

### 9.2.5 General statistical analysis methods

In general, all data collected will be listed. Categorical variables will be summarized by dose group as frequencies and percentages in each category. Continuous variables will be summarized by dose group as numbers of subjects, means, standard deviations, medians, and minimum/maximum values. Two-sided, 95% confidence intervals will be calculated for immunogenicity endpoints and differences between each dose group.

### 9.2.6 Baseline characteristics

Baseline characteristics will be listed and summarized and will include inclusion/exclusion criteria; demographics (e.g., age, sex, race/ethnicity); medical history.

### 9.2.7 Study drug administration

Details of study vaccine administration will be listed. In addition, a summary tabulation will be produced that shows the number of subjects in each group who received 1 dose.

### 9.2.8 Concomitant medications

Concomitant medications will be coded to generic terms using the World Health Organization Drug Dictionary (WHOdd). The listing will include dosing date,

WHOdd drug class, WHOdd preferred drug name, reported drug name, start and stop date, and whether the drug was taken after vaccination and up through Day 28 (± 2). The summary table will consist of numbers and percentages of subjects reporting each WHOdd drug class and preferred name within drug class.

### 9.2.9 Immunogenicity analysis

Immunogenicity parameters will be listed for all subjects and summarized and analyzed for the primary and secondary immunogenicity populations separately.

M2e and HAI specific IgG antibody titration curves will be established at each time point that data are available. At the start of the study volunteers will be scored as positive (IgG titers above an established negative baseline) or negative (IgG titers below the negative baseline) for pre-existing M2e or HAI specific IgG titers. The proportion of sero-negative volunteers who develop significantly positive antibody titers after immunization in each Group will be determined. The proportion of sero-positive volunteers who develop a significant rise in antibody titers after immunization in each Group will also be estimated. Standard statistical analyses will be used to distinguish significant changes in antibody titers. One-sided 95% lower confidence bound on the estimated proportion will also be constructed. All analyses will be based upon a per-protocol cohort with additional analysis performed for the intent-to-treat (ITT) cohort. The per protocol cohort is defined as all volunteers who complete both immunizations (VAX102 or placebo plus TIV).

Anti-M2e or HAI serum antibody titers will be listed and summarized for each time point that data are available. The mean change from baseline to Day 28 (± 2) in the log-transformed anti-M2e or HAI serum antibody reciprocal titers will be compared to zero within dose groups using t-tests. Analysis of variance (ANOVA) will be used to test for differences among dose groups, with Dunnett's test used to compare each dose group. Dose-response will be tested using vaccine and the amount of antigen in µg per group, coded as 1 µg or placebo. Pairwise comparisons will be used to evaluate differences between Groups. Proportion of subjects with an immune response at Days 14 (±2) and 28 (± 2) will be compared among Groups using a Fisher's exact test or chi square test of association.

### 9.2.10 Safety analysis

Safety parameters will be listed and summarized for all subjects in the safety population. Safety analysis will involve the examination of incidence and reasons for discontinuation; incidence of vaccination site abnormalities; incidence of other local and systemic AEs and their relationship to the study drug; and changes in clinical laboratory results, vital signs, and physical examination findings.

Reactogenicity will be analyzed using the severity grading in the Memory Aid and Targeted PE.

#### 9.2.10.1 Subject disposition

The primary reason for discontinuation from the study vaccine and/or the study will be listed and summarized.

#### 9.2.10.2 Reactogenicity and other AEs

Volunteered, observed, and elicited AEs will be recorded from the time of the vaccination through the Day 28 (± 2) follow-up visit AEs will be coded using MedDRA (Medical Dictionary for Regulatory Activities). In MedDRA, each reported event is mapped to a Lowest Level Term (LLT), a Preferred Term (PT), and a System Organ Class (SOC). A treatment-emergent AE will be defined as an AE that was not present prior to vaccination or was present but worsened in intensity or frequency after vaccination.

Tabulations of solicited AEs will be produced for immediate reactogenicity events that occur within the first 30 minutes of each vaccination, and separately for those that begin within 7 days of vaccination. All solicited reactions will be considered causally related to vaccination. Causality of all other AEs will be assessed by the investigator, as noted in **Sections 8.1.4** and **8.1.5**. AEs other than reactogenicity will be tabulated for several time periods: from baseline through Day 28 (± 2). Subject incidence will be summarized, therefore, each subject contributes only once to each preferred term or to a SOC. AE summaries will also be produced for events that are considered at least possibly related to study treatment, and for events of at least severe intensity. A statistical analysis for trend in increasing rates with increasing dose will be performed by the Cochran-Armitage trend test. Fisher's Exact test will be used to assess the significance of the difference in rates between the overall active vaccination group(s).

All reported AEs (treatment-emergent or not) will be listed. Listings will include study day, duration (days), relationship to treatment, severity, action taken, outcome, and whether or not serious criteria apply. Treatment-emergent AEs will be summarized, overall and by severity and relationship to treatment. For each SOC and PT within SOCs, the numbers and percentages of subjects reporting an event will be calculated. Similar listings and summaries will be done separately for reactogenicity and serious AEs.

#### 9.2.10.3 Clinical laboratory evaluation

Samples for chemistry and hematology parameters will be collected at screening and at follow-up visit on Day 1. Lab listings will include the study day, date and time collected. Tables of lab summaries will include means and mean changes from baseline for each time point at which lab data were collected. A separate series of tables will show numbers and percentages of subjects by out-of-range category (high, normal, low). Shift tables will be provided that show numbers and percentages of subjects that shift from one out-of-range category at baseline to another out-of-range category at selected subsequent time points.

#### 9.2.10.4 Vital signs

Vital signs (blood pressure, heart rate, respiratory rate, and temperature) will be obtained at screening, baseline, and at each follow-up visit. The listing will include the study day, date, and observed values. Summary tables will describe observed values and changes from baseline for each collection time.

#### 9.2.10.5 Physical examination and Injection Site Evaluation

Complete physical examinations will be performed at screening. A targeted physical examination of axillary lymph nodes will be performed at before and 30 minutes after vaccination on day 0, and again on day 1 (±1), day 14 (±2) and 28 (±2). Additional targeted physical examinations may be conducted if deemed warranted by the investigator. Clinically significant changes from baseline that meet the definition of an AE will be recorded as an AE. Summary tables will include numbers and percentages of subjects with abnormal findings for each body system.

# 10 CLINICAL STUDY MONITORING AND ADMINISTRATION

## 10.1 Informed Consent

Written informed consent must be obtained from each subject in accordance with FDA regulations set forth in Part 50 of Title 21 of the Code of Federal Regulations. Informed consent must be obtained prior to any study related procedures being performed.

The subject and person explaining informed consent must sign the current IRB-approved version of the consent form. A copy of the signed consent form will be given to the subject. The date that consent was obtained will be recorded on the CRF as well as in the subject's source document.

Original signed consents must be maintained at the site and be made available for inspection, as appropriate.

## 10.2 Study Documentation

A study binder or record must be maintained at the investigative site and must contain all essential regulatory documents, including a signed copy of the Investigator Agreement. VaxInnate or its representative will provide a Study Binder to the site.

According to Federal Regulations (21 CFR 312), all records related to this clinical trial must be retained by the Investigator for at least 2 years after the last approval of a marketing application and until there are no pending or contemplated marketing applications OR until at least 2 years have elapsed since the formal discontinuation of clinical development of the investigational product. The Sponsor will inform the Investigator as to when these documents no longer need to be retained. These documents must be stored in a safe location and be available in the event of a regulatory audit.

Study records that must be retained include, but are not necessarily limited to: subject charts or source documents, case report forms, investigational product disposition records, essential regulatory documents, and study reports as applicable.

## 10.3 Investigator Brochure

The current Investigator Brochure will be supplied to the investigator, as will any updates to the Investigator Brochure.

## 10.4 Confidentiality and Publication

The anonymity of subjects participating in this study must be maintained. Subjects will be identified by their assigned subject number and their initials (e.g. 001/XYZ) in all written communications between the Investigator and Sponsor. Documents that are not submitted to the Sponsor and that identify the subject (i.e. signed informed consent) will be made available to the Sponsor or regulatory authorities for inspection, but will be maintained in confidence.

All study related information provided by the Sponsor to the Investigator and not previously published, including but not limited to the study product identity, the Investigator's Brochure, the study protocol, verbal and written communication, CRFs, assay methods and scientific data, will be considered confidential. In addition, all information developed during the conduct of the clinical investigation of the study agent is also considered confidential. Neither the Investigator nor any of his/her employees or agents shall disclose or use this information for any purpose other than the performance of the clinical study. Such information shall remain the confidential and proprietary property of the Sponsor, and disclosure to others will be limited to other physicians who are conducting studies with the same study product, the IRB and the FDA except by prior written permission of the Sponsor or its agents. At such time that information becomes widely and publicly available through no fault of the Investigator, the obligation of nondisclosure toward that particular information will cease.

Initial publication of the results of this study will be of a cooperative nature that may include authors representing the Sponsor, Investigator(s), and collaborating scientists. Independent publications by involved individuals may follow.

At least 60 days prior to expected submission to the intended publisher or meeting committee, the Investigator must submit a copy of the desired presentation (oral or written) or publication manuscript to the Sponsor. This review period may be shortened upon mutual consent where circumstances require expeditious review. The Sponsor reserves the right to require modification of any publication, presentation or use by the Investigator if such activity may jeopardize a patent application, an existing patent or other proprietary rights. The Sponsor shall determine order of authorship of any publication combining all clinical results of this trial.

## 10.5 Institutional Review Board

The Investigator must submit the final protocol and proposed informed consent document to an Institutional Review Board (IRB) that is constituted and operates in accordance with Part 56 of Title 21 of the Code of Federal Regulations. The IRB will provide the Investigator with a written decision regarding the conduct of the study at that site and a copy of the document will be forwarded to the Project Manager. The study will not be initiated and no subjects will be enrolled until the appropriate documentation of IRB approval of the study protocol and the informed consent has been received.

Substantive modifications to the protocol will be submitted to the IRB for approval. These modifications may be implemented only after IRB written approval has been received and forwarded to the Project Manager. Administrative changes to the protocol such as a change that has no effect on the conduct of the study or risk to the subject should be submitted to the IRB for review, but formal approval is not required.

The Investigator must also submit any other written information that will be given to the study subjects as well as any advertisements for subject recruitment, if used, to the IRB for approval prior to implementing these documents.

The Investigator will make appropriate and timely reports to the IRB as required by applicable government regulations and IRB policy. In addition to progress reports, all known information regarding serious and unexpected adverse events, whether observed at their clinical site or at another site participating in a clinical investigation with the study product, will be reported to the IRB. It is the Sponsor and/or its representative’s responsibility to inform the Investigator of serious and unexpected events observed at other investigational sites.

It is the Investigator's obligation to provide the Sponsor and/or its representatives with copies of all study-related correspondence with the IRB in a timely fashion and to retain originals in a file. This IRB correspondence file will be made available as requested to appropriate representatives for monitoring or quality assurance review and to FDA representatives during site audits.

## 10.6 Ethical Study Conduct

This study is to be conducted in accordance with the ethical principles that originate in the Declaration of Helsinki.

## 10.7 Study Monitoring

So that the study may be adequately monitored, the Investigator will cooperate in providing the Sponsor's representatives with all study documents (e.g. subject charts and study files) and responding to inquiries that may arise as a result of the document review.

Review of these documents will usually occur during a routine monitoring visit, but may also be required during a visit by a quality assurance auditor. The Investigator will also provide access to these records to FDA representatives if and when requested. The Sponsor reserves the right to terminate the study if access to source documentation of work performed in this study is denied to the Sponsor or FDA representatives.

### 10.8 Quality Control and Quality Assurance

The Sponsor or designee will assure the accuracy of data, the selection of qualified Investigators, appropriate study centers and review protocol procedures with the Investigators and associated personnel prior to the study and during periodic monitoring visits. CRFs will be reviewed for accuracy and completeness by the Sponsor or a designee during on-site monitoring visits and after their return from the clinical site. Any discrepancies will be resolved with the Investigator as appropriate.

The study will be monitored by the Sponsor or its representatives as required by the FDA using the following methods:

- frequent telephone contacts
- periodic site visits
- review of original subject records, CRFs, investigational product accountability and storage, and general study documentation.

### 10.9 Protocol Amendments

Neither the Investigator nor the Sponsor will modify this protocol without obtaining the concurrence of the other. All protocol amendments must be signed and dated by the Investigator prior to implementation of the amendment. The Sponsor will submit protocol modifications to the FDA and other Regulatory Agencies as required. The Investigator is responsible for notifying the IRB of changes. Substantive changes will require IRB approval, such as changes in experimental procedures that affect subject safety, changes in dosage or study treatment, changes in assessment parameters, or changes in subject eligibility criteria.

In situations requiring a departure from the protocol, the Investigator or other physician in attendance will contact the Sponsor or designee by fax or telephone. If possible, this contact will occur before implementing any departure from protocol. In all cases, contact with the Sponsor or designee must be made as soon as possible in order to discuss the situation and agree on an appropriate course of action. The CRF and source documents must describe any departure from the protocol and the circumstances.

## 11 REFERENCES

Treanor JJ, Tierney EL, Zebedee SL, Lamb RA, Murphy BR. Passively transferred monoclonal antibody to the M2 protein inhibits influenza A virus replication in mice. J Virol, 1990. 64(3):1375-1377.

# APPENDIX A. SOLICITED LOCAL AND GENERAL AES

| Solicited local (injection site) AEs | Solicited general AEs |
|---|---|
| Redness | Fever |
| Swelling or induration | Headache |
| Pain | Fatigue |
| Ecchymosis | Joint pain |
| | Muscle aches |
| | Shivering (chills) |
| | Increased sweating |

Note: Oral temperature will be measured and recorded in the evening by the subject. Subjects will be instructed to take their temperatures at other times if they believe they are feverish. Should additional temperature measurements be performed at other times of day and recorded, the highest temperature in each 24-hour period will be entered into the eCRF.

# APPENDIX B. TOXICITY GRADING SCALE FOR HEALTHY ADULT AND ADOLESCENT VOLUNTEERS ENROLLED IN PREVENTIVE VACCINE CLINICAL TRIALS

## A Tables for Clinical Abnormalities

| Local Reaction to Injectable Product | Mild (Grade 1) | Moderate (Grade 2) | Severe (Grade 3) | Potentially Life Threatening (Grade 4) |
|---|---|---|---|---|
| Pain | Does not interfere with activity | Interferes with activity or repeated use of non-narcotic pain reliever | Prevents daily activity or repeated use of narcotic pain reliever | Emergency room (ER) visit or hospitalization |
| Tenderness | Mild pain to touch | Pain with movement | Significant pain at rest | ER visit or hospitalization |
| Erythema/Redness * | 2.5 - 5 cm | 5.1 - 10 cm | > 10 cm | Necrosis or exfoliative dermatitis |
| Swelling ** | 2.5 - 5 cm and does not interfere with activity | 5.1 - 10 cm or interferes with activity | > 10 cm or prevents daily activity | Necrosis |
| Ecchymosis * | 2.5 - 5 cm | 5.1 - 10 cm | > 10 cm | Necrosis |

* In addition to grading the measured local reaction at the greatest single diameter, the measurement should be recorded as a continuous variable.
** Swelling should be evaluated and graded using the functional scale as well as the actual measurement.

| Vital Signs * | Mild (Grade 1) | Moderate (Grade 2) | Severe (Grade 3) | Potentially Life Threatening (Grade 4) |
|---|---|---|---|---|
| Fever (°C) ** (°F) | 38.0 - 38.4 100.4 - 101.1 | 38.5 - 38.9 101.2 - 102.0 | 39.0 - 40 102.1 - 104 | > 40 > 104 |
| Tachycardia - beats per minute | 101 - 115 | 116 – 130 | > 130 | ER visit or hospitalization for arrhythmia |
| Bradycardia - beats per minute | 50 - 54 | 45 – 49 | < 45 | ER visit or hospitalization for arrhythmia |
| Hypertension (systolic) - mm Hg | 141 - 150 | 151 – 155 | >155 | ER visit or hospitalization for malignant hypertension |
| Hypertension (diastolic) - mm Hg | 91 - 95 | 96 – 100 | > 100 | ER visit or hospitalization for malignant hypertension |
| Hypotension (systolic) - mm Hg | 85 - 89 | 80 – 84 | < 80 | ER visit or hospitalization for hypotensive shock |
| Respiratory Rate - breaths per minute | 17 - 20 | 21 – 25 | > 25 | Intubation |

* Subject should be at rest for all vital sign measurements.
** Oral temperature; no recent hot or cold beverages or smoking.

| Systemic (General) | Mild (Grade 1) | Moderate (Grade 2) | Severe (Grade 3) | Potentially Life Threatening (Grade 4) |
|---|---|---|---|---|
| Nausea/ vomiting | No interference with activity or 1 - 2 episodes/24 hours | Some interference with activity or > 2 episodes/24 hours | Prevents daily activity, requires outpatient IV hydration | ER visit or hospitalization for hypotensive shock |
| Diarrhea | 2 - 3 loose stools or < 400 gms/ 24 hours | 4 - 5 stools or 400 - 800 gms/24 hours | 6 or more watery stools or > 800gms/24 hours or requires outpatient IV hydration | ER visit or hospitalization for hypotensive shock |
| Headache | No interference with activity | Some interference with activity or repeated use of non-narcotic pain reliever | Significant, prevents daily activity or repeated use of narcotic pain reliever | ER visit or hospitalization |
| Fatigue | No interference with activity | Some interference with activity | Significant, prevents daily activity | ER visit or hospitalization |
| Myalgia | No interference with activity | Some interference with activity | Significant, prevents daily activity | ER visit or hospitalization |
| Illness or clinical adverse event (as defined according to applicable regulation) | No interference with activity | Some interference with activity not requiring medical intervention | Prevents daily activity and requires medical intervention | ER visit or hospitalization |

## B Tables for Laboratory Abnormalities

| Serum chemistry | Mild (Grade 1) | Moderate (Grade 2) | Severe (Grade 3) | Potentially Life Threatening (Grade 4) |
|---|---|---|---|---|
| Blood Urea Nitrogen BUN mg/dL | 23 - 26 | 27 – 31 | > 31 | Requires dialysis |
| Creatinine – mg/dL | 1.1 - 1.5 | 1.6 – 2.0 | 2.1 - 2.5 | > 2.5 or requires dialysis |
| ALT increase by factor | 1.1 - 2.5 x ULN | 2.6 – 5.0 x ULN | 5.1 - 10 x ULN | > 10 x ULN |
| AST increase by factor | 1.1 - 2.5 x ULN | 2.6 – 5.0 x ULN | 5.1 - 10 x ULN | > 10 x ULN |
| Glucose - Hypoglycemia mg/dL | 65 - 69 | 55 – 64 | 45 – 54 | < 45 |
| Glucose - Hyperglycemia<br>Fasting - mg/dL<br>Random - mg/dL | 100 - 110<br>110 - 125 | 111 – 125<br>126 – 200 | >125<br>>200 | Insulin requirements or hyperosmolar coma |

* The laboratory values provided in the tables serve as guidelines and are dependent upon institutional normal parameters. Institutional normal reference ranges should be provided to demonstrate that they are appropriate.

| Hematology * | Mild (Grade 1) | Moderate (Grade 2) | Severe (Grade 3) | Potentially Life Threatening (Grade 4) |
|---|---|---|---|---|
| Hemoglobin (Female) - gm/dL | 12.0 - 13.0 | 10.0 - 11.9 | 8.0 - 9.9 | < 8.0 |
| Hemoglobin (Female) change from baseline value - gm/dL | Any decrease - 1.5 | 1.6 – 2.0 | 2.1 - 5.0 | > 5.0 |
| Hemoglobin (Male) – gm/dl | 12.5 - 14.5 | 10.5 - 12.4 | 8.5 - 10.4 | < 8.5 |
| Hemoglobin (Male) change from baseline value – gm/dL | Any decrease - 1.5 | 1.6 – 2.0 | 2.1 - 5.0 | > 5.0 |
| WBC Increase - cell/mm$^3$ | 10,800 - 15,000 | 15,001 - 20,000 | 20,001 - 25, 000 | > 25,000 |
| WBC Decrease - cell/mm$^3$ | 2,500 - 3,500 | 1,500 – 2,499 | 1,000 - 1,499 | < 1,000 |
| Lymphocytes Decrease - cell/mm$^3$ | 750 - 1,000 | 500 – 749 | 250 – 499 | < 250 |
| Neutrophils Decrease - cell/mm$^3$ | 1,500 - 2,000 | 1,000 – 1,499 | 500 – 999 | < 500 |
| Eosinophils - cell/mm$^3$ | 650 - 1500 | 1501 – 5000 | > 5000 | Hypereosinophilic syndrome |
| Platelets Decreased - cell/mm$^3$ | 125,000 - 140,000 | 100,000 - 124,000 | 25,000 – 99,000 | < 25,000 |

* The laboratory values provided in the tables serve as guidelines and are dependent upon institutional normal parameters. Institutional normal reference ranges should be provided to demonstrate that they are appropriate.

## Urinalysis

| Urine * | Mild (Grade 1) | Moderate (Grade 2) | Severe (Grade 3) | Potentially Life Threatening (Grade 4) |
|---|---|---|---|---|
| Protein | Trace | 1+ | 2+ | >2+ |
| Glucose | Trace | 1+ | 2+ | >2+ |
| Blood (microscopic) - red blood cells per high power field (rbc/hpf) | 1-10 | 11-50 | > 50 and/or gross blood | Hospitalization or packed red blood cells (PRBC) transfusion |

* The laboratory values provided in the tables serve as guidelines and are dependent upon institutional normal parameters. Institutional normal reference ranges should be provided to demonstrate that they are appropriate.

# APPENDIX C: SCHEDULE OF PROCEDURES AND ASSESSMENTS FOR VACCINATION

| **Procedure/ Assessment** | **Screen** | **Day 0** | **Day 0** | **Day 0** | **Day 1** | **Day 14** | **Day 28** | **Unsch visit** |
|---|---|---|---|---|---|---|---|---|
| **Range (days)** | **(-30 to -1)** | **(0)** | **(0)** | **(0)** | **(±1)** | **(±2)** | **(±2)** | |
| | | **Pre** | **Vac** | **30 min** | | | | |
| Informed Consent | X | | | | | | | |
| Review of I/E Criteria | X | X | | | | | | |
| Demographics | X | | | | | | | |
| Medical History | X | X | | | | | | |
| Physical Examination | X | | | | | | | |
| Urine Pregnancy Test | X | X | | | | | | |
| Urinalysis | X | | | | | | | |
| Vital Signs | X | X | | X | X | X | X | X |
| Blood for safety labs | X | | | | X | | | |
| Blood for CRP | | X | | | X | | | |
| Baseline Signs & Symptoms | | X | | | | | | |
| Vaccination (FLUVIRIN®) | | | X | | | | | |
| Vaccination (VAX102) | | | X | | | | | |
| Review of Memory Aid | | | | X | X | X | | |
| Review AE and con med | | | | X | X | X | X | X |
| Targeted Physical Exam | | X | | X | X | X | X | X |
| Injection Site Evaluation | | X | | X | X | X | X | |
| Blood Serology (M2e, HAI) | | X | | | | X | X | |
